# Standard language proficiency as social capital: Impacts on subjective socioeconomic status in China

**DOI:** 10.1371/journal.pone.0334861

**Published:** 2025-11-20

**Authors:** Yangyang Wei, Xin Cai

**Affiliations:** 1 School of Humanities and Communication, Zhejiang University of Finance and Economics, Hangzhou, China; 2 School of Statistics and Mathematics, Shanghai Lixin University of Accounting and Finance, Shanghai, China; Bahir Dar University, ETHIOPIA

## Abstract

Although language proficiency—especially in a standard language—has been widely studied as an indicator of social status and mobility, its relationship with subjective socioeconomic status (SES) requires closer examination. Drawing on data from the 2021 China General Social Survey, this study investigates the relationship between Mandarin proficiency and Chinese individuals’ subjective SES, including subjective class identity and perceptions of social mobility. The sample comprises 5,997 individuals aged 18–95 from urban and rural areas nationwide, with balanced sex distribution and diverse educational and occupational backgrounds. Mandarin proficiency is positively associated with static subjective class identity. This association is more pronounced among younger, less-educated, and rural groups. Additionally, proficiency alleviates the downward deviation, where subjective class identity falls below objective SES. This effect is stronger in coastal regions. However, it shows little association to perceptions of past or future mobility. Karlson-Holm-Breen (KHB) decomposition reveals that job and life satisfaction partially mediate the relationship, with life satisfaction exerting a stronger influence. These findings underscore the role of Mandarin proficiency as social capital, reflecting individuals’ perceptions of their social position. The findings also provide empirical evidence to inform state-led language standardization policies that promote social integration and reduce disparities.

## Introduction

Standard language is an institutionalized and codified language variety, characterized by standardized grammar, vocabulary, and pronunciation. It is used as the normative medium in formal domains, such as education, administration, and public communication [1]. In China, “standard language” refers specifically to Mandarin, which serves as the national linguistic norm. According to data released by the Ministry of Education of China in June 2022, the prevalence of Mandarin has increased from 70% to 80.72% since 2012 [2], highlighting the long-term effects of state-led language standardization in a linguistically diverse society.

Previous research has primarily focused on Mandarin proficiency as a stratified outcome, shaped by unequal access to material and institutional resources and reflecting entrenched patterns of the social hierarchy [3–7]. However, less attention has been paid to the reverse perspective—that is, how language proficiency itself acts as a factor influencing social stratification. Bourdieu’s theory offers a valuable lens for addressing this question. It conceptualizes standard language proficiency as a form of capital that determines one’s legitimacy and position in social interactions [8–10]. Crucially, his framework “recognizes agency, identity, and subjectivity” [11], and “allows a way of relating stratification theory to issues of identity and subjectivity” [12]. However, existing studies that treat Mandarin proficiency as an explanatory factor have primarily relied on objective socioeconomic status (SES), such as income, education, and occupation. As a result, they have overlooked a crucial question: how language proficiency shapes individuals’ subjective SES.

In response to this question, this study uses data from the 2021 China General Social Survey (CGSS), a comprehensive and nationally representative dataset that includes direct measures of subjective social status. This enables us to test the association between Mandarin proficiency and subjective SES and to examine the potential pathways and mechanisms that underlie this relationship. By integrating a subjective perspective and an analysis of mechanisms into the study of language and social stratification, this research offers a novel contribution and policy-relevant insights for multidialectal, socially diverse contexts.

### Literature review and research hypotheses

#### Standard language proficiency as social capital in stratified societies.

According to Bourdieu’s theory, the process of modern state-building has heightened the demand for proficiency in standard languages. The rise of standard languages reflects the transformation of linguistic practices into unequally distributed capital, thereby situating standard language proficiency at the core of stratification processes in nationalizing societies [8,9]. Building on this framework, it is essential to consider how standard language proficiency has been conceptualized in studies of social stratification.

Research in the economics of language largely views standard language proficiency as instrumental capital, which influences material aspects of social stratification by enhancing educational opportunities [13], employment prospects [14,15], and wealth accumulation [16–18]. By contrast, sociolinguistic studies emphasize standard language proficiency as a form of symbolic capital. It serves as a marker of legitimacy and social belonging in various domains, including education [19–21], the labor market [22,23], and public services [24–26]. Through such implicit mechanisms, it contributes significantly to the reproduction of social inequality.

Although the literature mentioned above has explored the relationship between standard language proficiency and social stratification from these perspectives, most studies primarily rely on objective SES indicators such as occupation, income, and education [27,28], overlooking a critical dimension of social stratification: subjective SES. As early as 1973, Jackman and Jackman emphasized that how individuals perceive their position within the social hierarchy is crucial for understanding social inequality [29]. Subsequent research has further suggested that subjective social status may be a more effective measure of individuals’ social position [30–32]. However, research on the relationship between standard language proficiency and subjective SES remains limited, which provides a key rationale for the present study.

#### Mandarin proficiency and subjective SES in contemporary China.

As a paradigmatic example of a rapidly developing country, China’s swift accumulation of wealth has coexisted with widening income and opportunity inequalities [33,34]. This persistent and widely perceived inequality has heightened public concern about individuals’ relative positions in the social hierarchy [35]. In the literature, subjective SES is commonly conceptualized along two dimensions: first, the static perception of one’s current SES, namely subjective class identity; and second, the dynamic perception of SES mobility, which encompasses retrospective evaluations of past mobility and expectations of future mobility [36].

In the Chinese context, subjective SES exhibits distinctive features. Empirical studies consistently show that individuals’ subjective class identity tends to be lower than their objective SES, reflecting relative deprivation and status dissatisfaction [36,37]. Simultaneously, owing to structural transformations, such as market reforms since the 1980s, the loosening of the household registration system, labor market expansion, and state-owned enterprise reforms [34,38], Chinese individuals have maintained strong and enduring perceptions of social mobility [39].

Existing studies have identified a range of socioeconomic conditions and social attitudes as significant determinants of subjective SES [40,41]. However, language factors—particularly standard language proficiency—have not yet been systematically examined, despite their potential role as a form of social capital. Moreover, unlike the bottom-up, market-driven processes of language standardization in Western societies, the Chinese case represents a top-down, state-led project [42]. While intended to promote national cohesion and communicative equality, the institutionalized gatekeeping mechanisms of this policy have produced “unanticipated” stratification effects through Mandarin proficiency [43,44]. Fluent speakers of Mandarin are often perceived as possessing instrumental and symbolic capital, enabling them to be better integrated into mainstream society, whereas those lacking such proficiency may risk marginalization [45–47]. Nevertheless, most existing studies remain theoretical or based on small-scale surveys, and systematic evidence from nationally representative data remains scarce.

Against this backdrop, the present study conceptualizes Mandarin proficiency as social capital and examines its role in shaping individuals’ static and dynamic perceptions of subjective SES. This perspective contributes to a more comprehensive understanding of the mechanisms underlying subjective SES and offers empirical insights for language policy and for fostering social integration in contemporary China.

#### Research hypotheses.

Based on the preceding theoretical framework and research gaps, this study formulates hypotheses to examine how Mandarin proficiency influences individuals’ subjective SES, focusing on two key dimensions: static and dynamic perceptions of SES.

The first dimension—static perceptions of SES—concerns individuals’ evaluations of their current position in the social hierarchy:

**Hypothesis 1.1.** Mandarin proficiency is positively associated with individuals’ subjective class identity.

**Hypothesis 1.2.** Higher Mandarin proficiency reduces the probability of downward deviation in subjective class identity.

The second dimension—dynamic perceptions of SES—includes both retrospective and prospective evaluations:

**Hypothesis 2.1.** Mandarin proficiency is negatively associated with individuals’ perceptions of downward class mobility in the past.

**Hypothesis 2.2.** Mandarin proficiency is negatively associated with individuals’ expectations of downward class mobility in the future.

Beyond the direct associations, this study considers potential mediating pathways. Prior studies suggest that subjective socioeconomic status is influenced by various social and psychological factors, such as perceived job control and satisfaction with living standards [48,49]. Sociolinguistic research further shows that language proficiency enhances job satisfaction [50,51] and life satisfaction [52–55]. Accordingly, this study considers these two factors as potential mediators linking Mandarin proficiency to perceptions of social status and proposes the following hypotheses:

**Hypothesis 3.1.** Mandarin proficiency enhances individuals’ job satisfaction, which in turn partially mediates the relationship between language proficiency and subjective SES.

**Hypothesis 3.2.** Mandarin proficiency enhances individuals’ life satisfaction, which in turn partially mediates the relationship between language proficiency and subjective SES.

**Fig 1:**
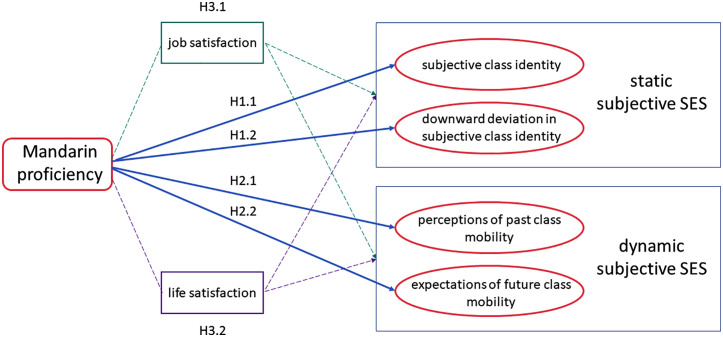
Conceptual framework: Pathways from Mandarin proficiency to subjective socioeconomic status.

Fig 1 illustrates the conceptual framework of this study, summarizing the hypothesized relationships.

## Research methodology

### Data sources and sample selection

This study draws on data from the 2021 CGSS, a nationally representative and large-scale survey. Notably, the CGSS includes self-reported Mandarin proficiency and subjective SES, which enables an investigation of the relationship between language proficiency and individuals’ perceived social status. The 2021 CGSS yielded 8,148 valid questionnaires. A total of 2,151 samples were excluded owing to missing data on key variables, primarily income. The final analytical sample consists of 5,997 individuals aged 18–95 from both urban and rural areas nationwide. The sample shows a balanced sex distribution and diverse educational and occupational backgrounds. To assess potential selection bias, we compared the excluded and retained samples on core demographic characteristics (age, sex, and education level). No significant differences were found, suggesting that the missing data are likely missing at random.

### Model specification

Consistent with established practices in empirical research [36,55], two types of models were employed. For the binary outcomes—downward deviation in class identity, perceived class Mobility, and expected class mobility—we employed probit models. For the ordinal outcome—subjective class identity, measured on a five-category scale—we used an ordered probit model.

The ordered probit model can be expressed as follows. Let y* denote the latent continuous variable representing subjective class identity:


$${\text{y}}^{*}=\alpha +\beta \text{mandarin}+\gamma \text{s}+\varepsilon ,\varepsilon \sim \text{N}(\text{0},\text{1}).$$ (1)

The observed ordinal outcome y is determined by threshold-crossing:


$$\begin{array}{c} y=j\;if\;{\;u}_{j-\text{1}}<y*\leq u_{j},j=\text{1},\text{2},\ldots ,\text{5}, \end{array}$$ (2)

where $u_{j}$ are the estimated thresholds. The probability of observing category j is:


$$\begin{array}{c} P(y=j)=\phi (u_{j}-X\beta )-\phi (u_{j-\text{1}}-X\beta ), \end{array}$$ (3)

with Φ(·) denoting the standard normal cumulative distribution function. Parameters and thresholds are estimated via Maximum Likelihood Estimation (MLE).

### Measurement of variables

#### Dependent variable.

This study employed the following dependent variables, which were derived from the 2021 CGSS. All variables were coded so that a higher score indicated a higher social class position.

(1) Individuals’ Subjective Class Identity: Measured by the self-reported question: “Overall, how would you classify your socioeconomic status?” Participants selected a category from a scale with explicit labels. Responses were reverse-coded to a numerical scale ranging from 1 (lower class) to 5 (upper class).
(2) Individuals’ Class Identity Deviation: Calculated as the difference between participants’ subjective class identity and objective SES. Following Li and Wang [56], objective SES was measured by the question “What was your total income last year (2020)?,” and categorized into five levels: 1=below half the sample average; 2=between half and the full average; 3=between one and three times the average; 4=between three and six times the average; and 5 = six times or more the average. Class identity deviation was computed as subjective class identity minus objective SES. If the value was negative—that is, subjective class identity was lower than objective SES—it was coded as 1 (downward deviation); otherwise, as 0 (no downward deviation).
(3) Individuals’ Perception of Class Mobility: Based on two questions: “Overall, where do you perceive yourself to be positioned within the social hierarchy in today’s society?” and “What level do you think you were in 10 years ago?” It was calculated as the current class rating minus the past rating. If the value was negative—that is, participants perceived their current class status as lower than it was 10 years ago—it was coded as 1 (perceived downward mobility); otherwise, it was coded as 0 (no perceived downward mobility).
(4) Individuals’ Expectation of Class Mobility: Based on two questions: “Overall, where do you perceive yourself to be positioned within the social hierarchy in today’s society?” and “What level do you think you will be in 10 years?” It was calculated as the current class rating minus the expected class rating. If the value was negative—that is, participants anticipated their future status to be lower than current status—it was coded as 1 (expected downward mobility); otherwise, it was coded as 0 (no expected downward mobility).

Prior studies have demonstrated that self-reported social status reliably reflects individuals’ perceived position in the hierarchy [29,32,57]. In the Chinese context, empirical research further confirmed the reliability of CGSS-based measures of subjective social status during a period of rapid transition [30,36].

#### Independent variable.

The independent variable in this study is participants’ self-reported Mandarin-speaking proficiency. This choice was guided by three primary considerations. First, prior research suggests that, compared to objective tests, self-reported measures may offer greater ecological validity and comparability when examining the relationship between language proficiency and individuals’ subjective perceptions. They directly reflect a person’s own experiences and self-evaluation [55]. Second, our focus on speaking ability, rather than listening or comprehension skills, was a deliberate methodological choice. Mandarin-speaking ability is a central component of the national Mandarin Proficiency Test and reflects an individual’s proactive engagement in communication. Third, because Mandarin-speaking ability is generally weaker than listening ability among Chinese citizens [3], choosing this weaker skill as the independent variable more effectively reveals the bottleneck effect of language limitations on SES.

Based on the question, “What level do you think your ability to speak Mandarin is?,” responses were coded from 1 (cannot speak at all) to 5 (very good). Higher scores indicate higher levels of Mandarin proficiency.

#### Mediator variables.

Building on Hypotheses 3.1 and 3.2, job satisfaction and life satisfaction were included as mediators. These variables were measured by the questions, “Overall, are you satisfied with your current job?” and “Overall, are you satisfied with your life?” Responses were rated on a five-point scale, with higher values indicating greater satisfaction.

#### Control variables.

Based on existing research on factors influencing individuals’ perceptions of SES and data completeness, we included 11 control variables: sex, age, education level, occupation type, marital status, personal annual income, vehicle ownership, social medical insurance, social pension insurance, provincial-level economic development, and urban-rural affiliation. Descriptive statistics for all variables are presented in Table 1.

**Table 1:** Variable description and descriptive statistics.

Variable Attribute	Variable Name	Mean	Description and Descriptive Statistics
Dependent Variable	Subjective Class Identity	2.30	1 = Lower Class (22.54%), 2 = Lower-Middle Class (32.13%), 3 = Middle Class (39.02%), 4 = Upper-Middle Class (5.92%), 5 = Upper Class (0.38%)
	Downward Deviation in Class Identity	–	0 = No Class Identity deviation (84.09%), 1 = Class Identity deviation (15.91%)
	Perception of Downward Mobility	–	0 = No Perception (85.96%), 1 = Perception of Downward Class Mobility (14.04%)
	Expectation of Downward Mobility	–	0 = No Expectation (90.95%), 1 = Expectation of Downward Class Mobility (9.05%)
Independent Variable	Speaking Proficiency in Mandarin	3.02	Max = 5, Min = 1, Standard Deviation = 1.140
Mediator Variable	Job Satisfaction	1.87	Max = 4, Min = 1, Standard Deviation = 0.843
	Life Satisfaction	3.98	Max = 5, Min = 1, Standard Deviation = 0.736
Control Variable	Sex	–	0 = Male (46.19%), 1 = Female (53.81%)
	Age	51.53	Max = 95, Min = 18, Standard Deviation = 17.00
	Education Level	5.42	Years of education (years), Max = 13, Min = 1, Standard Deviation = 3.337
	Occupation Type	–	0 = Unemployed (46.78%), 1 = Farming (15.71%), 2 = Non-Agricultural (37.82%)
	Marital Status	–	0 = Unmarried (26.90%), 1 = Married (73.10%)
	Personal Annual Income	7.84	Log-transformed after adding 0.01, Max = 16.11, Min = 2.30, Standard Deviation = 5.006
	Vehicle Ownership	–	0 = No Private Car (55.86%), 1 = Has Private Car (44.14%)
	Social Medical Insurance	–	0 = Not Participated (5.57%), 1 = Participated (94.43%)
	Social Pension Insurance	–	0 = Not Participated (25.95%), 1 = Participated (74.05%)
	Province GDP		Max = 11.739 (trillion), Min = 0.459 (trillion)
	Urban-Rural Affiliation	–	0 = Rural (67.83%), 1 = Urban (32.17%)

## Research findings

### Descriptive statistics

Table 2 presents the characteristics of the study sample, categorized by self-reported Mandarin-speaking proficiency. The distribution of Mandarin proficiency is relatively balanced, with 30.70% reporting “Good” to “Very good,” 37.68% reporting “Average,” and 31.53% reporting “Poor” or “Cannot speak at all.” Consistent with previous research, Mandarin-speaking proficiency is strongly correlated with demographic and socioeconomic factors. Participants with higher Mandarin proficiency tend to be younger, more highly educated, and have higher incomes.

**Table 2:** Individuals’ Mandarin proficiency and group characteristics.

Mandarin Proficiency Level	Proportion of respondents (%)	Age (Years)	Bachelor’s Degree or Above (%)	Annual Income (10,000 Yuan)	Subjective Class Identity	Downward Deviation in Class Identity (%)	Perception of Downward Mobility (%)	Expectation of Downward Mobility (%)
Cannot Speak at All	11.92	65.80	0.30	1.24	1.99	5.79	15.24	13.87
Poor	19.61	59.47	1.37	3.40	2.15	10.67	15.61	9.71
Average	37.68	49.33	9.21	3.61	2.30	17.12	13.16	7.89
Good	19.26	44.64	24.28	6.08	2.47	17.31	14.53	9.88
Very Good	11.44	42.68	39.16	4.83	2.51	27.50	12.41	5.92

Those self-reporting no Mandarin ability have the lowest subjective class identity (1.99), whereas participants with “Very good” proficiency score highest (2.51), exceeding the overall average of 2.30. Overall, this suggests a positive association between Mandarin proficiency and subjective class identity.

The relationship between Mandarin proficiency and other status perceptions reveals more nuanced patterns. The participants with “Very good” proficiency reported the highest rate of downward deviation in class identity (27.50%), compared to only 5.79% among those with no Mandarin ability. Conversely, those with higher Mandarin proficiency are less likely to perceive past downward class mobility and hold more optimistic expectations for future mobility. These descriptive findings suggest that Mandarin proficiency relates differently to various dimensions of subjective status perception. This highlights the need for further examination with inferential methods.

### Ordered probit regression

Table 3 reports the results of the Probit regression analysis on the relationship between Mandarin proficiency and perceptions of SES. Model (1) employs an ordered probit regression to investigate the relationship between Mandarin proficiency and subjective class identity. The results indicate a significant positive association: participants with higher Mandarin proficiency report a higher position in the social hierarchy; conversely, those with lower proficiency report lower position, supporting Hypothesis 1.1. To further illustrate the substantive implications, marginal effects were calculated. A one-unit increase in Mandarin proficiency reduces the probability of being in the lowest SES category by 3.50 percentage points (*dy/dx* = −0.035, p < 0.01) and the second-lowest category by 1.10 percentage points (*dy/dx* = −0.011, p < 0.01). Conversely, it increases the probability of belonging to the middle SES group by 3.15 percentage points (*dy/dx* = 0.032, p < 0.01), the higher SES group by 1.31 percentage points (*dy/dx* = 0.013, p < 0.01), and the highest SES group by 0.14 percentage points (*dy/dx* = 0.001, p < 0.01). These marginal effects clearly indicate that improvements in Mandarin proficiency primarily reduce the likelihood of being classified into lower subjective SES, while enhancing the probability of being placed in middle and higher subjective SES. This underscores Mandarin proficiency’s role as a facilitator of upward socioeconomic positioning.

**Table 3:** Mandarin proficiency and individuals’ subjective socioeconomic status.

Variable Name	Subjective Class Identity	Class Identity Downward Deviation	Perception of Downward Mobility	Expectation of Downward Mobility
Models	Model (I) Ordered Probit Regression	Model (II) Probit Regression	Model (III) Probit Regression	Model (IV) Probit Regression
Speaking Mandarin Proficiency Level	.122*** (.015)	−.095*** (.029)	.025 (.023)	−.004 (.026)
Women	.145*** (.029)	−.167*** (.054)	−.106** (.044)	−.073 (.050)
Age	−.013** (.006)	.014 (.011)	.049*** (.009)	.090*** (.012)
Age Squared	.0002*** (.00006)	−.000** (.000)	−.000*** (.000)	−.001*** (.000)
Education Level	.020*** (.006)	−.008 (.010)	.011 (.009)	.014 (.011)
Personal Income	.015*** (.003)	.830*** (.053)	−.016*** (.005)	.003 (.006)
Urban	.117*** (.035)	−.073 (.058)	−.069 (.053)	−.144** (.064)
Social Medical Insurance	.144** (.063)	−.096 (.138)	−.089 (.093)	−.235** (.105)
Social Pension Insurance	.082** (.036)	−.107 (.070)	−.098* (.054)	.032 (.064)
Own Car	.285*** (.030)	−.276*** (.056)	−.096** (.046)	−.020 (.053)
Farming	−.044 (.045)	.159 (.119)	−.078 (.068)	.118* (.071)
Non-Farming	.090** (.040)	.093 (.077)	−.185*** (.060)	−.062 (.071)
Marital Status	.168*** (.036)	−.139** (.066)	−.041 (.054)	−.060 (.062)
GDP	.019*** (.005)	.008* (.010)	.012*** (.008)	−.009 (.009)
Pseudo $R^{\text{2}}$	.035	.131	.024	.055

*Note: The numbers in parentheses are the standard errors. ***, **, and * represent significance at the 1%, 5%, and 10% levels, respectively.

Model (2) shows that higher Mandarin proficiency is associated with a lower likelihood of downward deviation, in which subjective class identity falls below objective SES, supporting Hypothesis 1.2.

Models (3) and (4) assessed the relationship between Mandarin proficiency and individuals’ perceptions and expectations of downward social mobility. The analysis revealed no statistically significant associations, providing no empirical support for Hypotheses 2.1 and 2.2. These findings suggest that, while Mandarin proficiency may be associated with static perceptions of SES, it does not show a significant association with dynamic perceptions.

### Endogeneity treatment: Instrumental variables method

The empirical analysis may be subject to an endogeneity issue arising from two-way causality. For children, higher SES provides greater access to Mandarin education; while for adults, higher SES facilitates broader social networks, which helps improve Mandarin proficiency [58]. Therefore, while SES is influenced by Mandarin proficiency, proficiency itself may also be affected by SES, leading to biased estimates. Because the dependent variable—subjective class identity—is a discrete ordered variable, we adopted the conditional mixed process (CMP) method proposed by Roodman [59] for instrumental variable estimation.

Two instrumental variables were selected. The first is derived from the question: “In the past year, how often did you read books, newspapers, or magazines in your spare time?” Responses were coded on a five-point scale (from 1 = “never” to 5 = “daily”). This variable serves as a proxy for individuals’ exposure to print literacy practices. Regular reading contributes to the development of vocabulary and organizational skills, which in turn enhance oral expression [55], while being less directly related to subjective SES.

Second, the highest educational attainment of the mother was considered. Many studies have found that parents’ educational backgrounds can influence the development of their offspring’s language proficiency [60]. Based on the question “What is the highest level of education your mother has attained?” the options were coded on a 13-point scale, ranging from 1 = “no formal education” to 13 = “postgraduate or above”.

The CMP uses seemingly unrelated regression (SUR) and maximum likelihood estimation (MLE) to estimate the model through a recursive system of equations. Estimation proceeds in two stages: first, the association between the instrumental variables and Mandarin proficiency was estimated; and second, the first-stage estimates were incorporated into the model. The exogeneity of Mandarin proficiency was assessed using the endogeneity test parameter atanhrho_12. The regression results are presented in Table 4.

**Table 4:** Instrumental variables regression: Mandarin proficiency and subjective class identity.

	First Stage	Second Stage
Mandarin speaking proficiency		0.26***
Average Reading Frequency	0.205***	
Mother’s Years of Education	0.191***	
atanhrho_12		−1.48***
Other Controls	Included	Included
LR chi^2^	1750.39***	

Note: The asterisks (***, **, and *) represent statistical significance at the 1%, 5%, and 10% levels, respectively. “Other Controls” indicates that other variables are controlled for in the model. LR chi^2^ represents the likelihood ratio chi-square statistic.

According to Zhang and Cheng [55], if the endogeneity test parameter atanhrho_12 from the second stage is not zero, the ordered probit model exhibits endogeneity, and the CMP provides more reliable estimates. Table 4 shows that the parameter atanhrho_12 in the second stage was −1.48, indicating that the CMP estimation was more appropriate. In addition, we conducted instrument validity and overidentification tests to further address exogeneity. The first-stage F-statistic was 211.89 (>10), confirming strong correlations between the instruments and Mandarin proficiency. The Sargan overidentification test yielded a statistic of 0.0033 (p = 0.95), suggesting that the instruments are exogenous and satisfy the exclusion restriction. After CMP correction, the coefficient for Mandarin proficiency was 0.205 and statistically significant, supporting a positive association with subjective class identity. The CMP method provides more accurate estimates by eliminating endogeneity bias, better capturing the true effect of Mandarin proficiency.

We subsequently re-estimated the impact of Mandarin proficiency on the downward deviation of individuals’ subjective class identity using an instrumental variable probit model (IV-Probit). The p-value of the Wald test was greater than 0.1, indicating no significant endogeneity. These results suggest that the conclusions are robust and reflect the actual impact of Mandarin proficiency on downward deviation in subjective class identity.

### Propensity score matching

Sample selection bias may persist in the regression results because participants from different objective class positions already exhibit significant differences in Mandarin proficiency levels. In other words, the observed association between Mandarin proficiency and subjective SES may partly reflect the influence of objective class status. To address this issue, we applied the propensity score matching (PSM) method to re-estimate the effects.

Specifically, participants were divided into two groups based on their Mandarin-speaking proficiency: Good (combining “Good” and “Very Good”) and Poor (combining “Average,” “Poor,” and “Cannot speak at all”). The former served as the treatment group and the latter as the control group. Covariates that potentially affect both Mandarin proficiency and subjective SES (i.e., all control variables included in the regression models) were balanced across the two groups to eliminate significant differences. Under these conditions, the causal relationship between Mandarin proficiency and subjective SES was examined through differences in subjective class identity and perceptions of class mobility.

Among common PSM approaches (nearest neighbor matching, kernel matching, and radius matching), kernel matching was selected. This method successfully reduced the standardized bias of all confounding variables for the four dependent variables to below 10%, ensuring no significant differences in mean values between the two groups. The balance check ensured effective control of sample selection bias. The results (Table 5) indicate that participants with higher Mandarin proficiency exhibited significantly higher subjective class identity and a lower probability of downward deviation, further validating Hypotheses 1.1 and 1.2. However, no significant relationship was found between Mandarin proficiency and perceptions or expectations of downward mobility, thus failing to validate Hypotheses 2.1 and 2.2.

**Table 5:** Propensity score matching: Mandarin proficiency and subjective socioeconomic status.

	High Mandarin Proficiency	Average or Low Mandarin Proficiency	Average Treatment Effect	Standard Error	T-value
Subjective Class Identity	2.484	2.207	.277	.036	7.63***
Downward Deviation in Class Identity	.327	.388	−.060	.021	−2.80**
Perception of Downward Mobility	.156	.142	.014	.014	1.00
Expectation of Downward Mobility	.077	.062	.022	.009	1.58

*Note:***, **, and * denote significance at the 1%, 5%, and 10% levels, respectively.

### Robustness check

We employed three methods to assess the robustness of the results. The first method involved alternative model specification. For subjective class identity, we replaced the ordered probit model with an ordered logistic model. The coefficients obtained were positive and highly significant, consistent with the ordered probit results, indicating that higher Mandarin proficiency is associated with higher subjective class identity.

Second, both Mandarin proficiency and subjective class status were dichotomized, with scores of 1–3 coded as 0, and scores of 4–5 coded as 1. A probit regression was then conducted. The results were consistent with the previous ordered probit regression, with the Mandarin proficiency coefficient being significantly positive. Thus, across different model specifications, the results consistently indicated that higher Mandarin proficiency is associated with higher subjective class identity.

Third, Mandarin listening proficiency may serve as a proxy for overall language ability. Therefore, we substituted it for the explanatory variable and conducted an ordered probit regression. The coefficient remained significantly positive, consistent with the previous results.

Overall, the results appear robust and reliable.

### Mechanism analysis: Mediation effect test

We further applied the Karlson-Holm-Breen (KHB) decomposition to examine the mechanisms linking Mandarin proficiency and subjective class identity. The results in Table 6 indicate that 27.94% of the effect of Mandarin proficiency on subjective class identity is explained by job and life satisfaction. Specifically, individuals with higher Mandarin proficiency are more likely to experience greater workplace satisfaction, reflecting their sense of social recognition and professional success, which enhances their positive identity within their social class. Simultaneously, in daily life, they tend to receive greater social acceptance and emotional support. Higher life satisfaction further contributes to a more positive evaluation of their social class.

**Table 6:** The mediating effects of job satisfaction and life satisfaction.

		KHB Decomposition	
		Subjective Class Identity	Class Identity Downward Deviation
Total Effect		.138***	−.129*
Mediation Effect	Job Satisfaction	.022**	–
	Life Satisfaction	.017**	−.012*
Contribution Rate		27.94%	16.26%

*Note: ***, **, and * denote significance at the 1%, 5%, and 10% levels, respectively.

In the analysis of downward deviation in class identity, the mediating effect of life satisfaction is −0.012, which is statistically significant. This indicates that Mandarin proficiency effectively reduces the likelihood of downward deviation in class identity by enhancing life satisfaction. However, job satisfaction, which primarily reflects contentment with one’s professional life, might not necessarily translate into a stronger identification with social class or alignment with one’s objective class position. This effect may be particularly limited in environments where social mobility is restricted.

### Heterogeneity analysis

Given potential group differences, this study conducted heterogeneity tests based on sex, age, educational attainment, and household registration locations. Group differences in the regression coefficients were examined using the seemingly unrelated estimation (SUEST) method. The results are summarized below:

First, regarding sex, no significant difference was found in the association between Mandarin proficiency and subjective class identity for men and women. This finding suggests that both sexs share similar developmental resources and hold relatively equal positions in gaining social recognition through language proficiency.

Regarding age, Mandarin proficiency was positively associated with subjective SES across all age groups. Nonetheless, its effect on reducing downward deviation of class identity was particularly evident among individuals under 30 years of age. Younger individuals benefit more from Mandarin proficiency as social capital: it facilitates career opportunities, social network expansion, and upward mobility, thereby strengthening their positive identification and sometimes elevating it beyond their objective SES. By contrast, middle-aged and older individuals tend to have a more stabilized social identity, making them less influenced by language proficiency.

In terms of educational attainment, the positive effect of Mandarin proficiency on subjective class identity was significantly more substantial among individuals with vocational high school education or below (*P* < 0.1). For these groups, Mandarin proficiency may compensate for limited formal education and enhance their career prospects and perceived SES. Among the highly educated, however, the marginal utility of Mandarin proficiency is weaker, as education itself already provides substantial human capital and socioeconomic advantages.

In terms of household registration, the positive effect of Mandarin proficiency on the subjective class identity was significantly stronger among rural than urban residents (*P* < 0.1), indicating its particular value in enhancing rural residents’ SES perceptions.

Regarding region, no significant inland–coastal difference was observed in the overall association between Mandarin proficiency and subjective class identity. However, for downward deviation of class identity, coastal residents benefited more: Mandarin proficiency is more strongly linked to greater self-evaluation stability and fewer negative perceptions of status, a pattern less evident inland.

In summary, Mandarin proficiency has a more substantial effect among younger individuals, those with lower educational levels, and rural residents, highlighting its role in early career development, social mobility among less-educated groups, and bridging urban-rural disparities. In coastal regions, it also helps mitigate negative self-assessments of status, underscoring the unique role of linguistic capital in stabilizing social identity in dynamic economies. These findings further emphasize the value of Mandarin proficiency as social capital, supporting language promotion and social integration policies.

## Discussion

Since Labov’s seminal work [61], sociolinguistic research has predominantly treated language proficiency as an outcome shaped by social stratification. This perspective has been criticized for insufficient attention to how linguistic practices themselves participate in the production and reinforcement of social stratification systems [12]. We draw on large-scale, nationally representative data to examine how Mandarin proficiency, as a form of social capital, is associated with individuals’ subjective social status, thereby providing a novel perspective on social stratification.

First, previous studies have primarily focused on the link between Mandarin proficiency and objective SES [14,15]. Our study complements this literature by revealing a significant positive association between Mandarin proficiency and subjective SES. This finding resonates with similar observations in other multilingual societies [62–65], which suggests a certain universality in the relationship between language capital and subjective social status. As a paradigmatic case of top-down language standardization, China provides detailed evidence of this broader mechanism: even though the state’s standard language policy was initially designed to promote greater equality, it has nevertheless produced the “unanticipated consequence” of reinforcing class hierarchies based on language proficiency, echoing Weng’s theoretical argument [42].

Second, the study finds that Mandarin proficiency is associated with a lower likelihood of downward deviation in class identity. This phenomenon, in which perceived status falls below objective SES, has been the focus of considerable attention among Chinese sociologists [36,37]. By incorporating language as a status-relevant resource, this study suggests an additional mechanism. Standard language proficiency may serve as a psychosocial resource by conveying symbolic inclusion and legitimacy within stratified institutions. Conversely, limited proficiency may be correlated with feelings of marginalization and perceptions of lower social status. This interpretation extends prior accounts that focus primarily on inequality and relative deprivation, highlighting the potential buffering role of language in stabilizing subjective status.

Third, using KHB decomposition, the analysis indicates that both job satisfaction and life satisfaction partly explain the association between Mandarin proficiency and subjective class identity. However, job satisfaction is not significantly related to downward deviation in class identity. This finding highlights the possibility that employment gains alone might not resolve persistent mismatches between objective and perceived status. By contrast, life satisfaction—as a broader reflection of well-being—is more strongly linked to reduced downward deviation. Taken together, the results call for a more integrative approach to sociolinguistic stratification research—one that addresses both material outcomes and the psychological processes through which individuals internalize their social position.

Fourth, the results show no significant association between Mandarin proficiency and perceptions of either past or future class mobility. While this finding may appear to diverge from previous research on the poverty-reducing effects of language promotion [58], it highlights a crucial distinction: language proficiency can enhance individuals’ economic conditions and social integration without necessarily shifting their mobility perceptions. A plausible explanation is the absence of structural opportunities, which weakens the role of linguistic capital in shaping dynamic status perceptions. A study of sugar plantation workers in Guyana similarly revealed that in rigid stratification systems, mastery of the standard language does not necessarily translate into perceived opportunities of upward mobility [66]. Comparable mismatches between linguistic capital and social mobility have been documented in other developing societies [67–69]. In contemporary China, survey evidence further indicates a rising awareness of structural inequality. Respondents increasingly attribute socioeconomic gaps to unequal opportunities rather than individual merit, and report declining expectations of future mobility [70,71]. Taken together, these findings underscore a broader point: the value of linguistic capital is constrained by structural rigidities, whether standardization is driven by top-down state policies, as in China, or by bottom-up processes, as in other contexts.

## Conclusion

This study investigated the relationship between Mandarin proficiency and Chinese individuals’ subjective SES. By combining nationally representative CGSS data with a focus on subjective socioeconomic perceptions, it introduces an innovative research approach into the sociolinguistic study of stratification. The findings show that Mandarin proficiency is associated with static perceptions of SES—specifically, consolidating subjective status and reducing perceived inconsistencies between self-assessed and objective SES—but not with perceptions of past or future mobility. Mediation analysis highlights that life satisfaction—rather than job satisfaction—plays a stronger role in shaping this relationship. Heterogeneity analysis indicates stronger effects among younger, less-educated, and rural groups. In coastal areas, Mandarin proficiency more clearly reduces cases in which subjective class identity falls below objective SES.

This study provides policy implications for state-led standard language promotion. As a form of social capital, standard language proficiency strengthens subjective status and reduces mismatches between subjective and objective class positions. To maximize its integrative impact, promotion efforts should prioritize the younger, rural, and less-educated groups who are most likely to benefit. Practical measures can be advanced along four dimensions: first, by lowering economic barriers through subsidized training programs; second, by improving accessibility via community-based initiatives tailored to local needs; third, by ensuring that language learning translates into concrete benefits by fostering both employment prospects and broader life satisfaction; and fourth, by acknowledging the potential limits of language policy: if the social capital embodied in Mandarin proficiency fails to translate into real mobility gains, promotion efforts risk latent resistance. Overall, these measures underscore the need to integrate language standardization with broader reforms that jointly enhance equity and inclusion.

### Limitations and prospects

This study is subject to several limitations. First, the measurement of Mandarin proficiency was based on participants’ self-reports of speaking ability, without objective assessments of other dimensions of language competence, which limits the comprehensiveness and accuracy of the measure. Future research could incorporate standardized language tests to enhance measurement precision and validity. Second, relying on cross-sectional data captures socioeconomic perceptions at a single point in time, without tracing their dynamics. Given the rapid social transformation in China, longitudinal data would provide valuable insights into how these perceptions evolve. Third, while this study is based on a nationally representative survey, it might not fully capture variations in language use and communicative environments at the micro level. Future research could build on these findings by integrating qualitative approaches or regional case studies to explore the nuances of micro-level language practices and perceptions.

## Supporting information

S1 File Data. (ZIP)
