# Supplementary material for: Standard language proficiency as social capital: Impacts on subjective socioeconomic status in China
Source: PLoS One. 2025 Nov 20;20(11):e0334861. doi: 10.1371/journal.pone.0334861 (PMC12633880; doi:10.1371/journal.pone.0334861)
Supplement: S1 File — (ZIP) [file pone.0334861.s001.zip › cgss2021stata/CGSS2021问卷.pdf]

# 中国综合社会调查(CGSS)
# 2021 年居民问卷

中国人民大学中国调查与数据中心
中国综合社会调查项目组

2021 年 6 月

## 关于中国综合社会调查（CGSS）的说明

中国综合社会调查（Chinese General Social Survey，CGSS），是中国第一个全国性、综合性、连续性的大型社会调查项目。目的是通过定期、系统地收集中国人与中国社会各个方面的数据，总结社会变迁的长期趋势，探讨具有重大理论和现实意义的社会议题，推动国内社会科学研究的开放性与共享性，为政府决策与国际比较研究提供数据资料。

中国综合社会调查由中国人民大学联合全国各地的学术机构共同执行。从 2003 年开始，每年对全国各地一万多户家庭进行抽样调查。经过严格的科学抽样，我们选中了您家作为调查对象。您的合作对于我们了解有关情况和制定社会政策，有十分重要的意义。为了获得准确的数据，请您依据实际情况，回答访问员提出的问题。如果因此而对您的生活和工作造成不便，我们深表歉意，请您理解和帮助我们的工作。

对问卷中问题的回答，没有对错之分，您只要根据平时的想法和实际情况回答就行。对于您的回答，我们将按照《中华人民共和国统计法》第一章第九条和第三章第二十五条的规定，对您所提供的所有信息绝对严格保密，并且只用于统计分析，请您不要有任何顾虑。我们在以后的科学研究、政策分析以及观点评论中发布的是大量问卷的信息汇总，而不是您个人、家庭的具体信息，不会造成您个人、家庭信息的泄漏。请您放心。

在________________（省/自治区/直辖市）的调查，由中国人民大学和________________（合作单位名称）联合进行。参与调查的所有督导员和访问员都佩戴有中国人民大学统一核发的证件，如果您对调查员的身份有任何疑问，欢迎您随时拨打电话：010-62510695-1008 进行核查。

希望您协助我们完成这次访问，谢谢您的合作。

中国人民大学中国调查与数据中心

2021 年

# 入户情况登记与受访者抽样

## 地址院落确认

**S1.请确认是否找到该地址：**

住户清单列表序号：【CAPI】住户清单列表序号
建筑物编号：【CAPI】建筑物编号
所在楼层：【CAPI】所在楼层
楼层住宅数量：【CAPI】楼层住宅数量
住户编号：【CAPI】住户编号
住宅地址：【CAPI】住宅地址
户主姓名：【CAPI】户主姓名

找到上述地址 ........................................................................1
未找到上述地址，并已确认该地址不存在 ............................2 → **问卷结束**

**S2a.受访者居住的社区类型：**

未经改造的老城区（街坊型社区） ......................................1
单一或混合的单位社区 ..........................................................2
保障性住房社区 ......................................................................3
普通商品房小区 ......................................................................4
别墅区或高级住宅区 ..............................................................5
新近由农村社区转变过来的城市社区
（村改居、村居合并或“城中村”） ...................................6
农村 ..........................................................................................7
其他（请注明：__________________） ................................8

**S2b.楼宇/院落情况（多选）：**

不存在以下情况 ......................................................................1
有保安或者其他人看守 ..........................................................2
有门禁 ......................................................................................3
农村住户大门上锁 ..................................................................4
有看门狗 ..................................................................................5
其他（请注明：____________________） ...........................6

## 第一次入户联系

**S3.请记录联系受访家户的时间：**

**S4.请记录联系受访家户的情况：**

该地址住户愿意接受访问 ......................................................1 → **跳问 S27**
该地址无法接触 ......................................................................2 → **跳问 S7**
该地址无人在家 ......................................................................3 → **跳问 S7**

该地址无人居住 ..........................................................................4 → **跳问 S7**
该住址住户拒访 ..........................................................................5
其他无法继续访问的情况（请注明：________________）.....6 → **跳问 S7**

**S5.请记录拒绝人的性别：**

男 ..........................................................................................1
女 ..........................................................................................2
无法判断 ..................................................................................3

**S6.请记录拒绝人的年龄：**

20 岁以下 ................................................................................1
20-29 岁 ..................................................................................2
30-39 岁 ..................................................................................3
40-49 岁 ..................................................................................4
50-59 岁 ..................................................................................5
60 岁及以上 ............................................................................6
无法判断 ..................................................................................7

## 第二次入户联系

**S7.请记录联系受访家户的时间：**

**S8.请记录联系受访家户的情况：**

该地址住户愿意接受访问 ..........................................................1 → **跳问 S27**
该地址无法接触 ..........................................................................2 → **跳问 S11**
该地址无人在家 ..........................................................................3 → **跳问 S11**
该地址无人居住 ..........................................................................4 → **跳问 S11**
该住址住户拒访 ..........................................................................5
其他无法继续访问的情况（请注明：________________）.....6 → **跳问 S11**

**S9.请记录拒绝人的性别：**

男 ..........................................................................................1
女 ..........................................................................................2
无法判断 ..................................................................................3

**S10.请记录拒绝人的年龄：**

20 岁以下 ................................................................................1
20-29 岁 ..................................................................................2
30-39 岁 ..................................................................................3
40-49 岁 ..................................................................................4
50-59 岁 ..................................................................................5
60 岁及以上 ............................................................................6
无法判断 ..................................................................................7

## 第三次入户联系

**S11.请记录联系受访家户的时间：**

**S12.请记录联系受访家户的情况：**

该地址住户愿意接受访问........................................................1 → **跳问 S27**
该地址无法接触..........................................................................2 → **跳问 S15**
该地址无人在家..........................................................................3 → **跳问 S15**
该地址无人居住..........................................................................4 → **跳问 S15**
该住址住户拒访..........................................................................5
其他无法继续访问的情况（请注明：______________） .....6 → **跳问 S15**

**S13.请记录拒绝人的性别：**

男..................................................................................................1
女..................................................................................................2
无法判断......................................................................................3

**S14.请记录拒绝人的年龄：**

20 岁以下....................................................................................1
20-29 岁.......................................................................................2
30-39 岁.......................................................................................3
40-49 岁.......................................................................................4
50-59 岁.......................................................................................5
60 岁及以上.................................................................................6
无法判断......................................................................................7

## 第四次入户联系

**S15.请记录联系受访家户的时间：**

**S16.请记录联系受访家户的情况：**

该地址住户愿意接受访问........................................................1 → **跳问 S27**
该地址无法接触..........................................................................2 → **跳问 S19**
该地址无人在家..........................................................................3 → **跳问 S19**
该地址无人居住..........................................................................4 → **跳问 S19**
该住址住户拒访..........................................................................5
其他无法继续访问的情况（请注明：______________） .....6 → **跳问 S19**

**S17.请记录拒绝人的性别：**

男..................................................................................................1
女..................................................................................................2
无法判断......................................................................................3

**S18.请记录拒绝人的年龄：**

20 岁以下……………………………………1
20-29 岁……………………………………2
30-39 岁……………………………………3
40-49 岁……………………………………4
50-59 岁……………………………………5
60 岁及以上…………………………………6
无法判断……………………………………7

# 第五次入户联系

**S19.请记录联系受访家户的时间：**

**S20.请记录联系受访家户的情况：**

该地址住户愿意接受访问……………………1 → **跳问 S27**
该地址无法接触………………………………2 → **跳问 S23**
该地址无人在家………………………………3 → **跳问 S23**
该地址无人居住………………………………4 → **跳问 S23**
该住址住户拒访………………………………5
其他无法继续访问的情况（请注明：_______________）.....6 → **跳问 S23**

**S21.请记录拒绝人的性别：**

男……………………………………………1
女……………………………………………2
无法判断……………………………………3

**S22.请记录拒绝人的年龄：**

20 岁以下……………………………………1
20-29 岁……………………………………2
30-39 岁……………………………………3
40-49 岁……………………………………4
50-59 岁……………………………………5
60 岁及以上…………………………………6
无法判断……………………………………7

# 第六次入户联系

**S23.请记录联系受访家户的时间：**

**S24.请记录联系受访家户的情况：**

该地址住户愿意接受访问........................................................1 → **跳问 S27**
该地址无法接触.......................................................................2 → **问卷结束**
该地址无人在家.......................................................................3 → **问卷结束**
该地址无人居住.......................................................................4 → **问卷结束**
该住址住户拒访.......................................................................5
其他无法继续访问的情况（请注明：______________）.....6 → **问卷结束**

**S25.请记录拒绝人的性别：**

男..........................................................................................1
女..........................................................................................2
无法判断.................................................................................3

**S26.请记录拒绝人的年龄：**

20 岁以下...............................................................................1 → **问卷结束**
20-29 岁..................................................................................2 → **问卷结束**
30-39 岁..................................................................................3 → **问卷结束**
40-49 岁..................................................................................4 → **问卷结束**
50-59 岁..................................................................................5 → **问卷结束**
60 岁及以上............................................................................6 → **问卷结束**
无法判断.................................................................................7 → **问卷结束**

# 户抽样

**S27.样本清单上是否标明了该地址的户主姓名？【访问员记录】**

是..........................................................................................1
否..........................................................................................2 → **跳问 S29**

**S28.请问是【CAPI】户主姓名家吗？**

是..........................................................................................1
否..........................................................................................2

【访员注意】若答话人回答“否”，则向答话人解释可能抽样信息有误，但仍要在该地址继续访问

**S29.请问这个地址上只有您一户住吗（一户包括一个家庭户或无家庭关系的多人共居一个房间）？**

是..........................................................................................1 → **跳问 S33**
否..........................................................................................2

**S30.我们只需要请其中一户接受访问。请问有多少户住在这里？**

[____|____]

**S31.请问每一户都怎样称呼？**

| | 称呼 |
|---|---|
| 1 | |
| 2 | |
| 3 | |

| | 称呼 |
|---|---|
| 4 | |
| 5 | |
| 6 | |
| 7 | |
| 8 | |
| 9 | |
| 10 | |

【访员注意】

1.若答话人住在该地址，请将答话人所属住户填写在第一行，其余住户依开门答话人所述依次填写，中间不能空行;

2.集体户，即无家庭关系的多人共居（如企业员工合租的宿舍），以每一个居住房间为单独一户;

3.家庭户和集体户混合居住在一个地址户内，则以家庭户为单独一户，集体户以每一个居住房间为单独一户;

4.如果超过10户，则只记录答话人随机提供的前10户，11户及后面的所有住户默认不进入抽样框。

**S32.【CAPI】if(S32>=1, S32, rand(1, count(S31_1, S31_2, S31_3, S31_4, S31_5, S31_6, S31_7, S31_8, S31_9, S31_10)))**

**S33.（我们抽到的是【CAPI】抽中家户这一户，）请问是家庭户还是集体户？**

家庭户 ........................................................................................1

集体户 ........................................................................................2

【访员注意】

1.家庭户是指同吃同住且一同承担其它家庭生活费用的家户;

2.集体户是指无家庭关系的多人共居。

## 受访者抽样

**S34.请问您这一户目前一共住了多少年满 18 岁的人（公历 2003 年 12 月 31 日及以前出生）？我指的是：在您这一户里居住了 7 天以上或将要居住 7 天以上的 18 岁以上的人，包括亲戚、保姆/室友等其他人在内。**

[____|____]

**0→问卷结束**

**S35.他们的姓名/称呼/称谓，以及他们的性别和年龄分别是：**

| | 姓名/称呼/称谓/与答话人的关系 | 性别：1. 男 2. 女 | 年龄 |
|---|---|---|---|
| 1 | ________________ | [____] | [____|____|____] |
| 2 | ________________ | [____] | [____|____|____] |
| 3 | ________________ | [____] | [____|____|____] |
| 4 | ________________ | [____] | [____|____|____] |
| 5 | ________________ | [____] | [____|____|____] |
| 6 | ________________ | [____] | [____|____|____] |

| | 姓名/称呼/称谓/与答话人的关系 | 性别：1. 男 2. 女 | 年龄 |
|---|---|---|---|
| 7 | ________________ | [___] | [___|___|___] |
| 8 | ________________ | [___] | [___|___|___] |
| 9 | ________________ | [___] | [___|___|___] |
| 10 | ________________ | [___] | [___|___|___] |

【访员注意】

1.如果答话人年满18岁且在户内居住了7天以上或将要居住7天以上，则将答话人填写在第一行;

2.当S34填写的数字大于10时，则按答话人回答顺序填写前10位;

3.依次填写，中间不能空行;

4.只填写年满18岁的中国大陆公民。

**S36.【CAPI】if(S36>= 1, S36, rand(1, count(S35_1_1, S35_2_1, S35_3_1, S35_4_1, S35_5_1, S35_6_1, S35_7_1, S35_8_1, S35_9_1, S35_10_1)))**

## 第一次联系受访者

**S37.**

抽中的受访者为：**【CAPI】选定调查对象的姓名或称谓**

性别：**【CAPI】选定调查对象的性别**

年龄：**【CAPI】选定调查对象的年龄**

**S38.我们选定的调查对象是【CAPI】选定调查对象的姓名或称谓，请问我现在能访问他/她吗？**

选定的调查对象愿意接受访问........................................1 → **跳问 A00**

选定的调查对象不在家........................................2

选定的调查对象拒绝访问........................................3

选定的调查对象生病/受伤........................................4

选定的调查对象无能力接受调查........................................5

选定的调查对象不存在........................................6 → **问卷结束**

其他无法继续访问的情况（请注明：______________） .....7

## 第二次联系受访者

抽中的受访者为：**【CAPI】选定调查对象的姓名或称谓**

性别：**【CAPI】选定调查对象的性别**

年龄：**【CAPI】选定调查对象的年龄**

**S39.请记录联系受访家户的时间：**

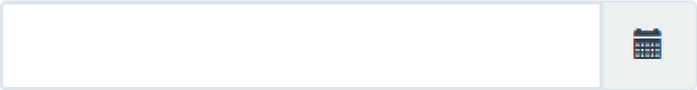

**S40.请记录联系受访家户的情况：**

该地址住户愿意接受访问........................................1 → **跳问 S43**

该地址无法接触........................................2 → **跳问 S44**

该地址无人在家........................................3 → **跳问 S44**

该地址无人居住......................................................................................4 → **跳问 S44**
该住址住户拒访......................................................................................5
其他无法继续访问的情况（请注明：______________） .....6 → **跳问 S44**

**S41.请记录拒绝人的性别：**
男..............................................................................................................1
女..............................................................................................................2
无法判断..................................................................................................3

**S42.请记录拒绝人的年龄：**
20 岁以下..................................................................................................1 → **跳问 S44**
20-29 岁....................................................................................................2 → **跳问 S44**
30-39 岁....................................................................................................3 → **跳问 S44**
40-49 岁....................................................................................................4 → **跳问 S44**
50-59 岁....................................................................................................5 → **跳问 S44**
60 岁及以上..............................................................................................6 → **跳问 S44**
无法判断..................................................................................................7 → **跳问 S44**

**S43.请记录联系受访者的情况：**
选定的调查对象愿意接受访问...........................................................1 → **跳问 A00**
选定的调查对象不在家........................................................................2
选定的调查对象拒绝访问....................................................................3
选定的调查对象生病/受伤...................................................................4
选定的调查对象无能力接受调查.........................................................5
选定的调查对象不存在........................................................................6 → **问卷结束**
其他无法继续访问的情况（请注明：______________） .....7

## 第三次联系受访者

抽中的受访者为：**【CAPI】选定调查对象的姓名或称谓**
性别：**【CAPI】选定调查对象的性别**
年龄：**【CAPI】选定调查对象的年龄**
**S44.请记录联系受访家户的时间：**

**S45.请记录联系受访家户的情况：**
该地址住户愿意接受访问....................................................................1 → **跳问 S48**
该地址无法接触......................................................................................2 → **跳问 S49**
该地址无人在家......................................................................................3 → **跳问 S49**
该地址无人居住......................................................................................4 → **跳问 S49**
该住址住户拒访......................................................................................5
其他无法继续访问的情况（请注明：______________） .....6 → **跳问 S49**

**S46.请记录拒绝人的性别：**

男........................................................................................1
女........................................................................................2
无法判断..............................................................................3

**S47.请记录拒绝人的年龄：**

20 岁以下............................................................................1 → **跳问 S49**
20-29 岁..............................................................................2 → **跳问 S49**
30-39 岁..............................................................................3 → **跳问 S49**
40-49 岁..............................................................................4 → **跳问 S49**
50-59 岁..............................................................................5 → **跳问 S49**
60 岁及以上........................................................................6 → **跳问 S49**
无法判断..............................................................................7 → **跳问 S49**

**S48.请记录联系受访者的情况：**

选定的调查对象愿意接受访问..........................................1 → **跳问 A00**
选定的调查对象不在家......................................................2
选定的调查对象拒绝访问..................................................3
选定的调查对象生病/受伤..................................................4
选定的调查对象无能力接受调查......................................5
选定的调查对象不存在......................................................6 → **问卷结束**
其他无法继续访问的情况（请注明：______________） .....7

## 第四次联系受访者

抽中的受访者为：**【CAPI】选定调查对象的姓名或称谓**
性别：**【CAPI】选定调查对象的性别**
年龄：**【CAPI】选定调查对象的年龄**
**S49.请记录联系受访家户的时间：**

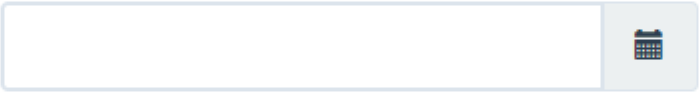

**S50.请记录联系受访家户的情况：**

该地址住户愿意接受访问..................................................1 → **跳问 S53**
该地址无法接触..................................................................2 → **问卷结束**
该地址无人在家..................................................................3 → **问卷结束**
该地址无人居住..................................................................4 → **问卷结束**
该住址住户拒访..................................................................5
其他无法继续访问的情况（请注明：______________） .....6 → **问卷结束**

**S51.请记录拒绝人的性别：**

男........................................................................................1
女........................................................................................2
无法判断..............................................................................3

**S52.请记录拒绝人的年龄：**

20 岁以下......................................................................................1 → **问卷结束**
20-29 岁........................................................................................2 → **问卷结束**
30-39 岁........................................................................................3 → **问卷结束**
40-49 岁........................................................................................4 → **问卷结束**
50-59 岁........................................................................................5 → **问卷结束**
60 岁及以上..................................................................................6 → **问卷结束**
无法判断........................................................................................7 → **问卷结束**

**S53.请记录联系受访者的情况：**

选定的调查对象愿意接受访问.................................................1
选定的调查对象不在家..............................................................2 → **问卷结束**
选定的调查对象拒绝访问..........................................................3 → **问卷结束**
选定的调查对象生病/受伤.........................................................4 → **问卷结束**
选定的调查对象无能力接受调查..............................................5 → **问卷结束**
选定的调查对象不存在..............................................................6 → **问卷结束**
其他无法继续访问的情况（请注明：______________）.....7 → **问卷结束**

# A 部分 核心模块

**A00.请记录当前时间：[____|____]月[____|____]日[____|____]时[____|____]分**

**A1.请问除您以外，您家里还有几个人？（注意！不要包括已经和您分家的成员）**

记录：[____|____]人

**A0101.请根据年龄大小告诉我们您家里每一个人的称呼（与被访者的关系）。**

| 家庭成员代码 | 家庭成员称呼（与被访者的关系） | **2.请问这些家庭成员和您的关系是？**<br>1 配偶　　7 祖父母/外祖父母<br>2 子女　　8 曾祖父母/曾外祖父母<br>3 父母　　9 孙子（女）/外孙子（女）<br>4 配偶的父母　　10 曾孙子（女）/曾外孙子（女）<br>5 兄弟姐妹　　11 其他（请注明：______）<br>6 女婿/儿媳 | **3.目前是否与您住在一起？**<br>1.住在一起<br>2.不住在一起 |
|---|---|---|---|
| **1** | 被访者本人 | | |
| **2** | | | |
| **3** | | | |
| **4** | | | |
| **5** | | | |
| **6** | | | |
| **7** | | | |
| **8** | | | |
| **9** | | | |
| **10** | | | |

访问员继续追问：还有哪些人现在您家常住，但没有出现在上表中

## 社会人口属性

**A2.性别【访问员记录】**

男 .................................................................................................. 1
女 .................................................................................................. 2

**A3.您的出生日期是什么？**

记录：[____|____|____|____]年[____|____]月[____|____]日

【访员注意】
1.记录公历年；
2.如果被访者以农历、生肖或其他方式报告自己的出生年，请换算成公历后再记录（可参照示卡1）；
3.如果被访者无法说清自己的出生日期，则以身份证或户口本日期为准；
4.年份必答，月和日不知道可填写 98，拒绝回答可填写 99。

**A4.您的民族是：**

汉 ........................................................................................ 1
蒙 ........................................................................................ 2
满 ........................................................................................ 3
回 ........................................................................................ 4
藏 ........................................................................................ 5
壮 ........................................................................................ 6
维 ........................................................................................ 7
其他（请注明：________________） .................................... 8

**A5.您的宗教信仰是什么？**

不信仰宗教................................................................................ 1
信仰宗教
佛教.......................................................................................... 11
道教.......................................................................................... 12
民间信仰（拜妈祖、关公等）.................................................. 13
回教/伊斯兰教.......................................................................... 14
天主教...................................................................................... 15
基督教...................................................................................... 16
东正教...................................................................................... 17
其他基督教.............................................................................. 18
犹太教...................................................................................... 19
印度教...................................................................................... 20
其他（请注明：________________） .................................... 21

**A6.您参加宗教活动的频繁程度是：**

从来没有参加过........................................................................ 1
一年不到 1 次............................................................................ 2
一年大概 1 到 2 次.................................................................... 3
一年几次.................................................................................... 4
大概一月 1 次............................................................................ 5
一月 2 到 3 次............................................................................ 6
差不多每周都有........................................................................ 7
每周都有.................................................................................... 8
一周几次.................................................................................... 9

**A7a.您目前的最高教育程度是：**

没有受过任何教育.................................................................... 1 → **跳问 A8a**
私塾、扫盲班............................................................................ 2 → **跳问 A8a**
小学.......................................................................................... 3
初中.......................................................................................... 4
职业高中.................................................................................... 5
普通高中.................................................................................... 6
中专.......................................................................................... 7
技校.......................................................................................... 8
大学专科（成人高等教育）...................................................... 9

大学专科（正规高等教育）........................................... 10
大学本科（成人高等教育）........................................... 11
大学本科（正规高等教育）........................................... 12
研究生及以上.................................................................... 13
其他（请注明：________________）...................................... 14

**A7b.您目前的最高教育程度是：**

正在读....................................................................................... 1
辍学和中途退学........................................................................ 2
肄业........................................................................................... 3
毕业........................................................................................... 4
不知道..................................................................................... 98
拒绝回答................................................................................. 99

**A7c.您<u>已完成的</u>最高学历是在哪一年获得的？**

记录：[____|____|____|____]年

【访员注意】
1.“已完成”指毕业；
2.9997.不适用、9998.不知道、9999.拒绝回答。

**A7d.若您上过高中，请问您所上高中的学校等级是什么？**

省/直辖市重点中学................................................................... 1
县、市级重点中学.................................................................... 2
区、乡/镇重点中学................................................................... 3
非重点中学................................................................................ 4
不分重点非重点........................................................................ 5
其他（请注明：________）................................................... 6

**【CAPI】若 A7a 选择“小学”、“初中”，则不回答该题**

**A7e.若您上过大学，请问您所上大学的学校等级是什么？**

中央或国家其他部委所属高等院校.......................................... 1
省属高等院校............................................................................ 2
地区所属高等院校.................................................................... 3
其他全日制高校........................................................................ 4
非全日制高校............................................................................ 5
其他（请注明：________）................................................... 6

**【CAPI】若 A7a 选择“小学”、“初中”、“职业高中”、“普通高中”、“中专”，不回答该题**

**A7f.若您上过大学，请问您最后所学专业（学科大类）是什么？**

哲学........................................................................................... 1
经济学....................................................................................... 2
法学........................................................................................... 3
教育学....................................................................................... 4
文学........................................................................................... 5
历史学....................................................................................... 6
理学........................................................................................... 7

工学........................................................................................ 8
农学........................................................................................ 9
医学........................................................................................ 10
军事学.................................................................................... 11
管理学.................................................................................... 12
艺术学.................................................................................... 13
其他（请注明：________） ................................................ 14

**【CAPI】若 A7a 选择“小学”、“初中”、“职业高中”、“普通高中”、“中专”，不回答该题**

**A8a.您个人去年（2020 年）全年的总收入是多少？**

[_______|_______|_______|_______|_______|_______|_______]

【访员注意】

1.高位补零；

2.9999996.个人全年总收入高于百万位数、9999997.不适用、9999998.不知道、9999999.拒绝回答。

**A8b.您个人去年（2020 年）全年的职业/劳动收入是多少？**

[_______|_______|_______|_______|_______|_______|_______]

【访员注意】

1.高位补零；

2.9999996.个人全年职业/劳动收入高于百万位数、9999997.不适用、9999998.不知道、9999999.拒绝回答。

**A9.请问，您递交过加入中国共产党的申请书吗？**

未递交过.................................................................................. 1
递交过，第一次递交申请书是[____|____|____|____]年......... 2
不知道...................................................................................... 98
拒绝回答.................................................................................. 99

【访员注意】9998.不知道、9999.拒绝回答

**A10.目前的政治面貌是：**

群众........................................................................................ 1
共青团员.................................................................................. 2
民主党派.................................................................................. 3
共产党员，入党时间是：[____|____|____|____]年................ 4
不知道...................................................................................... 98
拒绝回答.................................................................................. 99

【访员注意】9998.不知道、9999.拒绝回答

## 住房问题

**A11.您现在住的这座住房的套内建筑面积是：**[____|____|____|____]平方米

【访员注意】9998.不知道、9999.拒绝回答

**A12.您现在这座房子的产权（部分或全部产权）属于谁（多选）？**

自己所有.................................................................................. 1 → **跳问 A12b**

配偶所有...................................................................................... 2
子女所有...................................................................................... 3
父母所有...................................................................................... 4
配偶父母所有.............................................................................. 5
子女配偶所有.............................................................................. 6
其他家人/亲戚所有...................................................................... 7
家人/亲戚以外的个人或单位所有，这房是租（借）来的..... 8
其他情况（请注明：________________）............................ 9
不知道.................................................................................... 98
拒绝回答................................................................................ 99

**A12a.目前您是否拥有（包括与他人共同拥有）房产（拥有产权）？**

是 ............................................................................................ 1
否 ............................................................................................ 2 → **跳问 A13**
不知道.................................................................................... 98
拒绝回答................................................................................ 99

**A12b.目前您总共拥有几处房产（包括与他人共同拥有）：**

记录：[____|____|____]处

【访员注意】998.不知道、999.拒绝回答

**A12c.您（目前拥有的）各处房产的位置在哪？**

1. ________省/自治区/直辖市_________市+县/区
2. ________省/自治区/直辖市_________市+县/区
3. ________省/自治区/直辖市_________市+县/区
4. ________省/自治区/直辖市_________市+县/区
5. ________省/自治区/直辖市_________市+县/区

【访员注意】

1.不包括境外房产；

2.98.不知道、99.拒绝回答。

## 健康

**A13.您目前的身高是：**[____|____|____]厘米

【访员注意】998.不知道、999.拒绝回答

**A14.您目前的体重是：**[____|____|____]斤

【访员注意】

1.单位是斤（500 克），不是公斤（1000 克）；

2.998.不知道、999.拒绝回答。

**A15.您觉得您目前的身体健康状况是：**

很不健康...................................................................................... 1
比较不健康.................................................................................. 2
一般.............................................................................................. 3
比较健康...................................................................................... 4

很健康......................................................................................... 5
不知道....................................................................................... 98
拒绝回答..................................................................................... 99

**A16.在过去的四周中，由于健康问题影响到您的工作或其他日常活动的频繁程度是：**

总是............................................................................................. 1
经常............................................................................................. 2
有时............................................................................................. 3
很少............................................................................................. 4
从不............................................................................................. 5
不知道....................................................................................... 98
拒绝回答..................................................................................... 99

**A17.在过去的四周中，您感到心情抑郁或沮丧的频繁程度是：**

总是............................................................................................. 1
经常............................................................................................. 2
有时............................................................................................. 3
很少............................................................................................. 4
从不............................................................................................. 5
不知道....................................................................................... 98
拒绝回答..................................................................................... 99

## 迁移

**A18.您目前的户口登记状况是：**

农业户口 ....................................................................................... 1 → **跳问 A21**
非农业户口 ................................................................................... 2
居民户口（以前是农业户口） ...................................................... 3 → **跳问 A19a**
居民户口（以前是非农业户口） .................................................. 4
军籍 ............................................................................................... 5
没有户口 ....................................................................................... 6 → **跳问 A25**
其他（请注明：________________________）.......................... 7 → **跳问 A21**

**A19.您（目前）的非农户口是哪一年获得的？**

记录：[____|____|____|____]年

【访员注意】9997.自最初/出生就是、9998.不知道、9999.拒绝回答

**【CAPI】A18 选 2，并且 A19 填 9997（自出生就是非农业户口），则填答完 A19，跳问 A21**

**A19a.您是哪一年转为居民户口的？**

记录：[____|____|____|____]年

【访员注意】9997.自出生就是、9998.不知道、9999.拒绝回答

**【CAPI】A18 选 3、4 则填答本题，并且 A19a 填 9997（自出生就是居民户口），则填答完 A19a，跳问 A21**

**A20.您获得非农户口的途径是什么？**

升学........................................................................................ 1
参军........................................................................................ 2
工作（招工等）.......................................................................... 3
转干........................................................................................ 4
征地（包括村改居）..................................................................... 5
家属随转（包括通过婚姻）............................................................ 6
购买，或者通过购房.................................................................... 7
户口改革，当地不再有农业户口...................................................... 8
其他（请注明：________________________）.......................... 9
不知道...................................................................................... 98
拒绝回答................................................................................... 99

**A21.您目前的户口登记地是：**

本乡（镇、街道）........................................................................ 1 → **跳问 A24**
本县（市、区）其他乡（镇、街道）.............................................. 2 → **跳问 A24**
本区/县/县级市以外....................................................................... 3
户口待定.................................................................................... 4 → **跳问 A25**

**A22.您目前的户口登记地是：**

记录：__________省/自治区/直辖市___________市+县/区

【访员注意】97.境外、98.不知道、99.拒绝回答

**A23.您是哪一年离开户口登记地的？**

记录：[____|____|____|____]年 → **跳问 A25**

【访员注意】9996.在本地只是短期居住，如探亲或学生放暑假回家、9997.从未在户口登记地生活、9998.不知道、9999.拒绝回答

**A24.您的户口是哪一年迁到<u>本地（本区/县/县级市）</u>的？（请将具体年份填写在横线上）**

记录：[____|____|____|____]年

【访员注意】9997.自出生起一直就在本地→ **跳问 A25**、9998.不知道、9999.拒绝回答

**A24a.您的户口是<u>从哪里</u>迁到本地（本区/县/县级市）的？**

户口迁出地：__________省/自治区/直辖市___________市+县/区

【访员注意】97.境外、98.不知道、99.拒绝回答

**A25.您是哪一年来到本地（本区/县/县级市）居住的？**

记录：[____|____|____|____]年

【访员注意】9996.在本地只是短期居住，如探亲或学生放暑假回家、9997.自出生起一直就住在本地→ **跳问 A27a**、9998.不知道、9999.拒绝回答

**A26.您是从哪里（常住地）来到本地（本区/县/县级市）居住的？**

记录：__________省/自治区/直辖市__________市+县/区

【访员注意】97.境外、98.不知道、99.拒绝回答

**A27a.您出生时母亲的主要居住地是：**

记录：__________省/自治区/直辖市__________市+县/区

【访员注意】97.境外、98.不知道、99.拒绝回答

**A27b.您出生时母亲的常居地属于：**

农村........................................................................................ 1
乡镇........................................................................................ 2
县城........................................................................................ 3
城郊........................................................................................ 4
城市市区.................................................................................. 5
境外........................................................................................ 6
其他（请注明：__________________________）....................... 7
不知道.................................................................................... 98
拒绝回答................................................................................ 99

**A27c.您出生时的户口登记地是？**

记录：__________省/自治区/直辖市__________市+县/区

【访员注意】
1.97.境外、98.不知道、99.拒绝回答；
2.如果受访者出生时没有户口，则记录其首次有户口时的登记地。

**A27d.您出生时的户口登记地属于：**

农村........................................................................................ 1
乡镇........................................................................................ 2
县城........................................................................................ 3
城郊........................................................................................ 4
城市市区.................................................................................. 5
境外........................................................................................ 6
其他（请注明：__________________________）....................... 7
不知道.................................................................................... 98
拒绝回答................................................................................ 99

【访员注意】如果受访者出生时没有户口，则记录其首次有户口时的登记地

**A27e.您 14 周岁时的常居地是？**

记录：__________省/自治区/直辖市__________市+县/区

【访员注意】97.境外、98.不知道、99.拒绝回答

**A27f.您 14 周岁时的常居地属于：**

农村........................................................................................ 1
乡镇........................................................................................ 2
县城........................................................................................ 3
城郊........................................................................................ 4
城市市区.................................................................................. 5
境外........................................................................................ 6
其他（请注明：__________________________）....................... 7
不知道.................................................................................... 98

拒绝回答......................................................................................99

**A27g.您 14 周岁时的户口登记地是？**

记录：__________省/自治区/直辖市__________市+县/区

【访员注意】

1.97.境外、98.不知道、99.拒绝回答；

2.如果受访者 14 周岁时没有户口，则记录其首次有户口时的登记地。

**A27h.您 14 周岁时的户口登记地属于：**

农村..............................................................................................1
乡镇..............................................................................................2
县城..............................................................................................3
城郊..............................................................................................4
城市市区......................................................................................5
境外..............................................................................................6
其他（请注明：________________________）........................7
不知道........................................................................................98
拒绝回答......................................................................................99

【访员注意】如果受访者 14 周岁时没有户口，则记录其首次有户口时的登记地

**A27i.自 14 周岁开始，您总共在城市里居住过多少年（合计）？**

记录：[____|____]年

【访员注意】

1.向上取整；

2.96.从未在城市居住、97.一直在城市居住、98.不知道、99.拒绝回答。

**A27j.自 14 周岁开始，您总共在本地（本区/县/县级市）居住过多少年（合计）？**

记录：[____|____]年

【访员注意】

1.向上取整；

2.96.只是因旅游、出差等事宜短期居住，从未长期居住、97.一直在本地居住、98.不知道、99.拒绝回答。

## 生活方式

**A28.过去一年，您对以下媒体的使用情况是：【出示示卡 2】**

| | 从不 | 很少 | 有时 | 经常 | 非常频繁 | 不知道 | 拒绝回答 |
|---|---|---|---|---|---|---|---|
| **1. 报纸** | 1 | 2 | 3 | 4 | 5 | 98 | 99 |
| **2. 杂志** | 1 | 2 | 3 | 4 | 5 | 98 | 99 |
| **3. 广播** | 1 | 2 | 3 | 4 | 5 | 98 | 99 |
| **4. 电视** | 1 | 2 | 3 | 4 | 5 | 98 | 99 |
| **5. 互联网（包括手机上网）** | 1 | 2 | 3 | 4 | 5 | 98 | 99 |
| **6. 手机定制消息** | 1 | 2 | 3 | 4 | 5 | 98 | 99 |

**【CAPI】如果全部选择 1 → 跳问 A30**

**A29.在以上媒体中，哪个是您最主要的信息来源？**

报纸........................................................................................ 1
杂志........................................................................................ 2
广播........................................................................................ 3
电视........................................................................................ 4
互联网（包括手机上网）............................................... 5
手机定制消息....................................................................... 6
不知道.................................................................................. 98
拒绝回答.............................................................................. 99

**A30.过去一年，您是否经常在空闲时间从事以下活动？【出示示卡 3】**

| | 每天 | 一周数次 | 一月数次 | 一年数次或更少 | 从不 | 不知道 | 拒绝回答 |
|---|---|---|---|---|---|---|---|
| **1.** 看电视或者看碟 | 1 | 2 | 3 | 4 | 5 | 98 | 99 |
| **2.** 出去看电影 | 1 | 2 | 3 | 4 | 5 | 98 | 99 |
| **3.** 逛街购物 | 1 | 2 | 3 | 4 | 5 | 98 | 99 |
| **4.** 读书/报纸/杂志 | 1 | 2 | 3 | 4 | 5 | 98 | 99 |
| **5.** 参加文化活动（比如听音乐会，看演出和展览） | 1 | 2 | 3 | 4 | 5 | 98 | 99 |
| **6.** 与不住在一起的亲戚聚会 | 1 | 2 | 3 | 4 | 5 | 98 | 99 |
| **7.** 与朋友聚会 | 1 | 2 | 3 | 4 | 5 | 98 | 99 |
| **8.** 在家听音乐 | 1 | 2 | 3 | 4 | 5 | 98 | 99 |
| **9.** 参加体育锻炼 | 1 | 2 | 3 | 4 | 5 | 98 | 99 |
| **10.** 现场观看体育比赛 | 1 | 2 | 3 | 4 | 5 | 98 | 99 |
| **11.** 做手工（比如刺绣、木工） | 1 | 2 | 3 | 4 | 5 | 98 | 99 |
| **12.** 上网 | 1 | 2 | 3 | 4 | 5 | 98 | 99 |

**A30a.**您有没有归自己单独使用的手机？

有 ........................................................................................ 1
没有........................................................................................ 2
不知道.................................................................................. 98
拒绝回答.............................................................................. 99

**A30b.**在最近半年，您上过网吗，包括使用电脑、手机、智能穿戴等各种设备上网？

上过........................................................................................ 1
没上过.................................................................................... 2
不知道.................................................................................. 98
拒绝回答.............................................................................. 99

**A30c.**在最近半年，您家里其他的人上过网吗，包括使用电脑、手机、智能穿戴等各种设备上网？

上过........................................................................................ 1
没上过..................................................................................... 2
不知道..................................................................................... 98
拒绝回答................................................................................. 99

**A31.在过去一年中，您是否经常在您的空闲时间做下面的事情？【出示示卡 2】**

| | 从不 | 很少 | 有时 | 经常 | 非常频繁 | 不知道 | 拒绝回答 |
|---|---|---|---|---|---|---|---|
| **1.社交/串门** | 1 | 2 | 3 | 4 | 5 | 98 | 99 |
| **2.休息放松** | 1 | 2 | 3 | 4 | 5 | 98 | 99 |
| **3.学习充电** | 1 | 2 | 3 | 4 | 5 | 98 | 99 |

**A31a.请问您与邻居进行社交娱乐活动（如互相串门，一起看电视，吃饭，打牌等）的频繁程度是：**

几乎每天................................................................................. 1
一周 1 到 2 次......................................................................... 2
一个月几次............................................................................. 3
大约一个月 1 次..................................................................... 4
一年几次................................................................................. 5
一年 1 次或更少..................................................................... 6
从来不..................................................................................... 7
不知道..................................................................................... 98
拒绝回答................................................................................. 99

**A31b.请问您与其他朋友进行社交娱乐活动（如互相串门，一起看电视，吃饭，打牌等）的频繁程度是：**

几乎每天................................................................................. 1
一周 1 到 2 次......................................................................... 2
一个月几次............................................................................. 3
大约一个月 1 次..................................................................... 4
一年几次................................................................................. 5
一年 1 次或更少..................................................................... 6
从来不..................................................................................... 7
不知道..................................................................................... 98
拒绝回答................................................................................. 99

**A32.在过去一年中，您有多少个晚上是因为<u>出去度假或者探亲访友</u>而没有在家过夜？**

从未......................................................................................... 1
1-5 个晚上 .............................................................................. 2
6-10 个晚上 ............................................................................ 3
11-20 个晚上 .......................................................................... 4
21-30 个晚上 .......................................................................... 5
超过 30 个晚上........................................................................ 6
不知道..................................................................................... 98
拒绝回答................................................................................. 99

## 社会态度

**A33.总的来说，您同不同意在这个社会上，绝大多数人都是可以信任的？**

非常不同意.......................................................................... 1
比较不同意.......................................................................... 2
说不上同意不同意.............................................................. 3
比较同意.............................................................................. 4
非常同意.............................................................................. 5
不知道................................................................................ 98
拒绝回答............................................................................ 99

**A34.总的来说，您同不同意在这个社会上，您一不小心，别人就会想办法占您的便宜？**

非常不同意.......................................................................... 1
比较不同意.......................................................................... 2
说不上同意不同意.............................................................. 3
比较同意.............................................................................. 4
非常同意.............................................................................. 5
不知道................................................................................ 98
拒绝回答............................................................................ 99

**A35.总的来说，您认为当今的社会公不公平？**

完全不公平.......................................................................... 1
比较不公平.......................................................................... 2
说不上公平但也不能说不公平.......................................... 3
比较公平.............................................................................. 4
完全公平.............................................................................. 5
不知道................................................................................ 98
拒绝回答............................................................................ 99

**A36.总的来说，您觉得您的生活是否幸福？**

非常不幸福.......................................................................... 1
比较不幸福.......................................................................... 2
说不上幸福不幸福.............................................................. 3
比较幸福.............................................................................. 4
非常幸福.............................................................................. 5
不知道................................................................................ 98
拒绝回答............................................................................ 99

**A37.如果没有政策限制的话，您希望有几个孩子？**

希望有几个孩子 [____|____]
其中，希望有几个儿子 [____|____]
其中，希望有几个女儿 [____|____]

【访员注意】97.无所谓、98.不知道、99.拒绝回答

**A38.您认为婚前性行为对不对？**

总是不对的........................................................................ 1
大多数情况下是不对的............................................................ 2
说不上对与不对.................................................................... 3
有时是对的........................................................................ 4
完全是对的........................................................................ 5
不知道............................................................................ 98
拒绝回答.......................................................................... 99

**A39.您认为婚外性行为对不对？**

总是不对的........................................................................ 1
大多数情况下是不对的............................................................ 2
说不上对与不对.................................................................... 3
有时是对的........................................................................ 4
完全是对的........................................................................ 5
不知道............................................................................ 98
拒绝回答.......................................................................... 99

**A40.您认为同性间的性行为对不对？**

总是不对的........................................................................ 1
大多数情况下是不对的............................................................ 2
说不上对与不对.................................................................... 3
有时是对的........................................................................ 4
完全是对的........................................................................ 5
不知道............................................................................ 98
拒绝回答.......................................................................... 99

**A41.您认为有子女的老人的养老主要应该由谁负责？**

主要由政府负责.................................................................... 1
主要由子女负责.................................................................... 2
主要由老人自己负责................................................................ 3
政府/子女/老人责任均摊 ........................................................... 4
不知道............................................................................ 98
拒绝回答.......................................................................... 99

**A42.您是否同意以下说法：【出示示卡 4】**

| | 完全不同意 | 比较不同意 | 无所谓同意不同意 | 比较同意 | 完全同意 | 不知道 | 拒绝回答 |
|---|---|---|---|---|---|---|---|
| **1.** 男人以事业为重，女人以家庭为重 | 1 | 2 | 3 | 4 | 5 | 98 | 99 |
| **2.** 男性能力天生比女性强 | 1 | 2 | 3 | 4 | 5 | 98 | 99 |
| **3.** 干得好不如嫁得好 | 1 | 2 | 3 | 4 | 5 | 98 | 99 |
| **4.** 在经济不景气时，应该先解雇女性员工 | 1 | 2 | 3 | 4 | 5 | 98 | 99 |
| **5.** 夫妻应该均等分摊家务 | 1 | 2 | 3 | 4 | 5 | 98 | 99 |

## 阶层认同

**A43.在我们的社会里，有些人处在社会的上层，有些人处在社会的下层。这张卡片【出示示卡 5】的梯子要从上往下看。最高“10 分”代表最顶层，最低“1 分”代表最底层。**

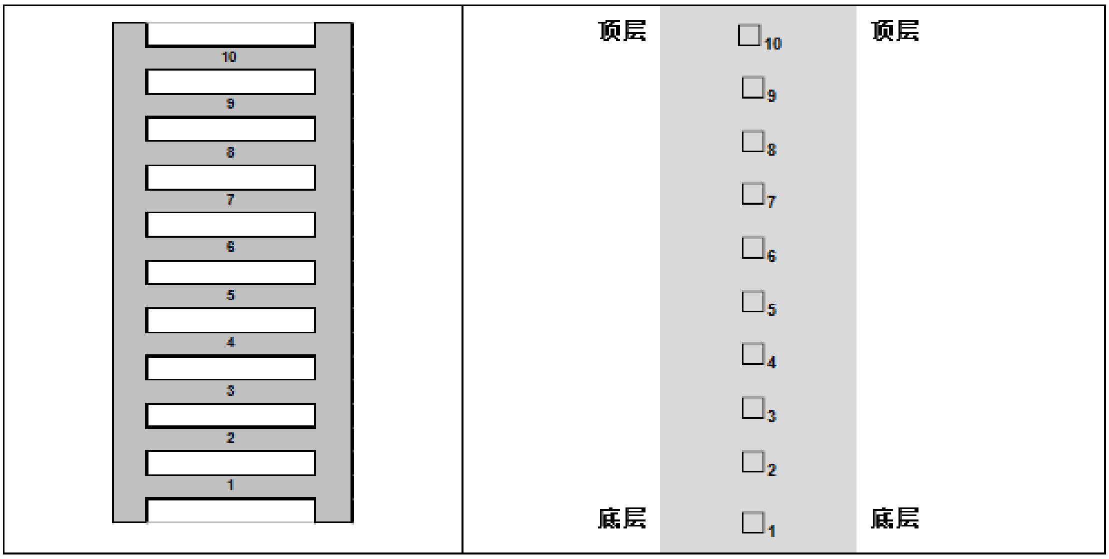


| | 1 分 | 2 分 | 3 分 | 4 分 | 5 分 | 6 分 | 7 分 | 8 分 | 9 分 | 10 分 | 不知道 | 拒绝回答 |
|---|---|---|---|---|---|---|---|---|---|---|---|---|
| **a.** 综合看来，在目前这个社会上，您本人处于社会的哪一层？ | 1 | 2 | 3 | 4 | 5 | 6 | 7 | 8 | 9 | 10 | 98 | 99 |
| **b.** 您认为您 10 年前在哪个等级上？ | 1 | 2 | 3 | 4 | 5 | 6 | 7 | 8 | 9 | 10 | 98 | 99 |
| **c.** 您认为您 10 年后将会在哪个等级上？ | 1 | 2 | 3 | 4 | 5 | 6 | 7 | 8 | 9 | 10 | 98 | 99 |
| **d.** 您认为在您 14 岁时，您的家庭处在哪个等级上？ | 1 | 2 | 3 | 4 | 5 | 6 | 7 | 8 | 9 | 10 | 98 | 99 |

**A43e.综合看来，在目前这个社会上，您本人的社会经济地位属于：**

上层........................................................................ 1
中上层..................................................................... 2
中层........................................................................ 3
中下层..................................................................... 4
下层........................................................................ 5
不知道..................................................................... 98
拒绝回答.................................................................. 99

## 政治参与行为与态度

**A44.上次居委会选举/村委会选举，您是否参加了投票？**

是 ........ 1
否 ........ 2
没有投票资格........ 3
不知道........ 98
拒绝回答........ 99

**A45.请问您是不是工会会员？**

是 ........ 1
以前是，现在不是........ 2
从来都不是........ 3
不知道........ 98
拒绝回答........ 99

**A46.如果有人在公共场所发布批评政府的言论，政府不应该干涉。您同意吗？【出示示卡 4】**

完全不同意........ 1
比较不同意........ 2
无所谓同意不同意........ 3
比较同意........ 4
完全同意........ 5
不知道........ 98
拒绝回答........ 99

**A47.生多少孩子是个人的事，政府不应该干涉。您同意吗？【出示示卡 4】**

完全不同意........ 1
比较不同意........ 2
无所谓同意不同意........ 3
比较同意........ 4
完全同意........ 5
不知道........ 98
拒绝回答........ 99

**A48.在哪里工作和生活是个人的自由，政府不应该干涉。您同意吗？【出示示卡 4】**

完全不同意........ 1
比较不同意........ 2
无所谓同意不同意........ 3
比较同意........ 4
完全同意........ 5
不知道........ 98
拒绝回答........ 99

## 个体认知能力

**A49.您觉得自己听普通话的能力是什么水平？**

完全听不懂................................................................................. 1
比较差........................................................................................... 2
一般............................................................................................... 3
比较好........................................................................................... 4
很好............................................................................................... 5
不知道........................................................................................... 98
拒绝回答....................................................................................... 99

**A50.您觉得自己说普通话的能力是什么水平？**

完全不能说................................................................................. 1
比较差........................................................................................... 2
一般............................................................................................... 3
比较好........................................................................................... 4
很好............................................................................................... 5
不知道........................................................................................... 98
拒绝回答....................................................................................... 99

**A51.您觉得自己听英语的能力是什么水平？**

完全听不懂................................................................................. 1
比较差........................................................................................... 2
一般............................................................................................... 3
比较好........................................................................................... 4
很好............................................................................................... 5
不知道........................................................................................... 98
拒绝回答....................................................................................... 99

**A52.您觉得自己说英语的能力是什么水平？**

完全不能说................................................................................. 1
比较差........................................................................................... 2
一般............................................................................................... 3
比较好........................................................................................... 4
很好............................................................................................... 5
不知道........................................................................................... 98
拒绝回答....................................................................................... 99

## 劳动力市场

**A53.您上一周是否为了取得收入而从事了一小时以上的劳动（包括参军）？**

未从事任何以获得经济收入为目的的工作.............................. 1 → **跳问 A54**
带薪休假，学习、临时停工或季节性歇业等.......................... 2
停薪休假，学习、临时停工或季节性歇业等.......................... 3
是，一般每周的工作时间[____|____|____]小时...................... 4 → **跳问 A57b**

**A53a.在您有工作的时候，通常情况下，您一周的工作时间大概有多少个小时，包括加班时间？**

记录：[____|____|____]小时 → **跳问 A57b**

【访员注意】

1.向上取整；

2.998.不知道、999.拒绝回答。

**A54.您上一周没有工作的原因是什么？**

在校学习........................................................................................ 1

丧失劳动能力.................................................................................. 2

毕业后未工作.................................................................................. 3

因单位原因失去原工作.................................................................. 4

因个人原因失去原工作.................................................................. 5

承包土地被征用.............................................................................. 6

离/退休 ........................................................................................... 7

料理家务........................................................................................ 8

其他（请注明：_______________） ....................................... 9

不知道.......................................................................................... 98

拒绝回答...................................................................................... 99

**A55.到目前为止，您连续没有工作的时间有多少个月？**

记录：[____|____|____]月

【访员注意】

1.向上取整；

2.997.从未工作过、998.不知道、999.拒绝回答。

**A56.您在最近三个月内采取过以下哪些方式寻找工作？（多选）**

未找过工作...................................................................................... 1

在职业介绍机构登记求职.............................................................. 2

委托亲友找工作.............................................................................. 3

利用网络及其他媒体求职.............................................................. 4

参加招聘会、或者自己直接上门询问.......................................... 5

为自己经营做准备.......................................................................... 6

其他（请注明：_______________） ....................................... 7

不知道.......................................................................................... 98

拒绝回答...................................................................................... 99

**【CAPI】未找过工作、不知道、拒绝回答不能与其他选项同时选择**

**A57a.如有适合的工作，您能否在两周内开始工作？**

能 .................................................................................................... 1

不能................................................................................................ 2

不知道.......................................................................................... 98

拒绝回答...................................................................................... 99

**A57b.您人生第一份工作的职业是什么？**

农林牧渔业劳动者.......................................................................... 1

非技术工人（体力工人） ............................................................. 2

技术工人（体力工人）........................................................ 3
商业、服务业人员............................................................... 4
一般办公室人员.................................................................. 5
中层管理人员...................................................................... 6
高级管理人员、单位负责人................................................ 7
一般专业技术人员............................................................... 8
中级专业技术人员............................................................... 9
高级专业技术人员............................................................... 10
私营企业主.......................................................................... 11
个体户.................................................................................. 12
军人或警察.......................................................................... 13
学生...................................................................................... 14
退休...................................................................................... 15
无业/失业 ............................................................................ 16
其他（请注明：__________________________）...................... 17
不知道.................................................................................. 98
拒绝回答.............................................................................. 99

【访员注意】"学生"选项仅适用于受访者目前是学生且从未有过正式工作

**A57c.您的第一份工作是在哪一年获得的？**

记录：[____|____|____|____]年

【访员注意】9997. 不适用、9998.不知道、9999.拒绝回答

**A57d.下列各种情形，哪一种更符合您第一份工作的状况？**

受雇于他人（有固定雇主）...................................................1
全职务农...............................................................................2
兼业务农，同时从事一些非农工作.......................................3
劳务工/劳动派遣人员............................................................4
零工、散工（无固定雇主的受雇者）...................................5
在自己家的生意或企业中工作/帮忙，领工资........................6
在自己家的生意或企业中工作/帮忙，不领工资....................7
自由职业者...........................................................................8
个体工商户...........................................................................9
自己是老板（或者是合伙人）.............................................10
其他（请注明：____________________）.......................................88
无法回答...............................................................................98

**A57e.您第一份工作的具体职业名称是：**

[________________________________________________________]

【访员注意】97.不适用、98.不知道、99.拒绝回答

**A58.您的工作经历及状况是？**

目前从事非农工作............................................................... 1
目前务农，曾经有过非农工作............................................. 2 → **跳问 A60a**
目前务农，没有过非农工作................................................. 3 → **跳问 A61**
目前没有工作，而且只务过农............................................. 4 → **跳问 A61**

目前没有工作，曾经有过非农工作........................................ 5 → **跳问 A60a**
从未工作过................................................................................ 6 → **跳问 A61**

**A58a.您目前是否同时兼有多份工作？**

没有........................................................................................ 1 → **跳问 A59a**
有，一共兼有[____|____]份工作 ............................................... 2
不知道...................................................................................... 98
拒绝回答.................................................................................. 99

【访员注意】98.不知道、99.拒绝回答

**A58b.那您目前最主要的工作大约占您职业收入的百分之多少？**

记录：[____|____|____]%

【访员注意】0.最主要的工作是务农→ **跳问 A61**、998.不知道、999.拒绝回答

访员注意：以下 A59 题的所有问题，如被访者有多份工作，则指的是他认为最主要的工作。

**A59a.下列各种情形，哪一种更符合您目前的工作的状况？**

自己是老板（或者是合伙人）................................................ 1
个体工商户.............................................................................. 2
受雇于他人（有固定雇主）.................................................... 3 → **跳问 A59b**
劳务工/劳务派遣人员................................................................ 4 → **跳问 A59b**
零工、散工（无固定雇主的受雇者）.................................... 5 → **跳问 A59c**
在自己家的生意/企业中工作/帮忙，领工资........................... 6 → **跳问 A59b**
在自己家的生意/企业中工作/帮忙，不领工资....................... 7 → **跳问 A59c**
自由职业者.............................................................................. 8 → **跳问 A59c**
其他（请注明：________________）.......................................... 9 → **跳问 A59b**
不知道...................................................................................... 98
拒绝回答.................................................................................. 99

**A59a1.请问您雇有的雇员数是？**

记录：[____|____|____|____|____]人 → **跳问 A59c**

【访员注意】99996.雇有人数超过五位数、99998.不知道、99999.拒绝回答

**A59b.您目前的工作是否与用人单位或雇主签订了书面劳动合同？**

没有签订劳动合同.................................................................... 1
签有无固定期限劳动合同........................................................ 2
签有固定期限劳动合同，期限为[____|____|____]月.............. 3
不知道...................................................................................... 98
拒绝回答.................................................................................. 99

【访员注意】998.不知道、999.拒绝回答

**A59c.从您第一份非农工作到您目前的工作，您一共工作了多少年？**

记录：[____|____]年

【访问注意】
1.向上取整；
2.98.不知道、99.拒绝回答。

**A59d1.您目前工作的具体职业名称是：**

[________________________________________________]

【访员注意】98.不知道、99.拒绝回答

**A59d2.您目前的具体工作内容是：**

[________________________________________________]

[________________________________________________]

【访员注意】98.不知道、99.拒绝回答

**A59e.您目前工作的性质是？**

全职工作.................................................................................. 1
非全职工作................................................................................ 2 → **跳问 A59f**
不知道.................................................................................... 98
拒绝回答.................................................................................. 99

**A59e1.您如果担任了行政职务，您的行政级别是：**

没有担任任何行政职务..............................................................0
无级别...........................................................................................1
股级...............................................................................................2
副科级...........................................................................................3
正科级...........................................................................................4
副处级...........................................................................................5
正处级...........................................................................................6
副司局级及以上...........................................................................7
不知道.........................................................................................98
拒绝回答.....................................................................................99

**A59f.在您目前的工作中，您的管理活动情况是:**

只管别人，不受别人管理........................................................ 1
既管理别人，又受别人管理..................................................... 2
只受别人管理，不管理别人..................................................... 3 → **跳问 A59g**
既不管理别人，又不受别人管理.............................................. 4 → **跳问 A59g**
不知道...................................................................................... 98
拒绝回答.................................................................................. 99

**A59f1.请问您管理的人数是：**

记录：[____|____|____|____|____]人

【访员注意】

1.管理人数指的是直接管理的人数；

2.99996.管理人数超过五位数、99998.不知道、99999.拒绝回答。

**A59g.在您目前的工作中，您在多大程度上能自主决定您工作的具体方式:**

完全自主决定........................................................................... 1
能在一定程度上自主................................................................. 2
在很少程度上自主..................................................................... 3

完全不能自主......4
不知道......98
拒绝回答......99

**A59h.在您目前的工作岗位上，是否经常有人希望通过您的工作便利帮他/她办事？**

总是......1
经常......2
有时......3
很少......4
从没有......5
不知道......98
拒绝回答......99

**A59i1.您目前工作的单位或公司的具体名称（全称）是：**

[________________________________]

【访员注意】98.不知道、99.拒绝回答

**A59i2.您目前工作的单位或公司的主要产品或服务是：**

[________________________________]
[________________________________]

【访员注意】98.不知道、99.拒绝回答

**A59j.您目前工作的单位或公司的单位类型是：**

党政机关......1 → **跳问 A59L**
企业......2
事业单位......3
社会团体、居/村委会......4
无单位/自雇（包括个体户）......5 → **跳问 A59m**
军队......6 → **跳问 A59m**
其他（请注明：__________）......7
不知道......98
拒绝回答......99

**A59k.您目前工作的单位或公司的所有制性质是：**

国有或国有控股......1
集体所有或集体控股......2
私有/民营或私有/民营控股......3
港澳台资或港澳台资控股......4
外资所有或外资控股......5
其他（请注明：__________）......6
不知道......98
拒绝回答......99

**A59L.您目前工作的单位或公司有多少员工？**

记录：[____|____|____|____|____]人

【访员注意】99996.员工人数超过五位数、99998.不知道、99999.拒绝回答

**A59m.到目前为止，您在目前的工作单位或公司工作了多少年？**

记录：[____|____]年 → **跳问 A61**

【访员注意】向上取整；98.不知道、99.拒绝回答

**【CAPI】当 A59a 选 3、4、5 并回答 L1 至 L21 题后，跳问 A61**

## 工作时间

**【CAPI】当 A59a 选 3、4、5 时回答该模块 L1 至 L21 题**

**L1.您目前的工资计算方式主要是？**

计件.....................................................................................1
计时.....................................................................................2
计件和计时都有.....................................................................3
按天计算...............................................................................4
底薪加提成（或底薪加绩效）.................................................5
固定月薪制............................................................................6
年薪制...................................................................................7
其他（请注明：________）....................................................8
不知道...................................................................................98
拒绝回答...............................................................................99

**L2.您平时从家（或住的地方）到工作单位的通勤时间（单程）是多少时间？**

[____|____|____]分钟

【访员注意】997.不适用、998.不知道、999.拒绝回答

**L3.您过去一个月工作了几天？**

[____|____]天

【访员注意】97.不适用、98.不知道、99.拒绝回答

**L4a.在过去的一个月内，您工作时间最长的一周，大概工作了多少小时？**

[____|____|____]小时

【访员注意】997.不适用、998.不知道、999.拒绝回答

**L4b.在过去的一个月内，您工作时间最短的一周，大概工作了多少小时？**

[____|____|____]小时

【访员注意】997.不适用、998.不知道、999.拒绝回答

**L5.您觉得一周工作时间超过多少小时算加班？**

[____|____|____]小时

【访员注意】997.不适用、998.不知道、999.拒绝回答

**L6.如果加班的话，加班后您更符合下列哪种情形？**

加班时间可以抵消请假时间...........................................1
领取加班工资..............................................................2
领取加班工资且可以抵消请假时间.................................3
无任何补偿..................................................................4

其他......................................................................................................5
不知道..................................................................................................98
拒绝回答..............................................................................................99

**L7.您过去一个周是否有加过班？**

加过班，且加班时间超过了调休时间........................................1
加过班，且加班时间与调休时间持平........................................2
加过班，但没有调休...............................................................3
没有加班..............................................................................4 → **跳问 L11**
不知道..................................................................................98
拒绝回答..............................................................................99

**L8.您过去一周共加班：**

[____|____|____]小时

**其中有报酬的有：**

[____|____|____]小时

【访员注意】997.不适用、998.不知道、999.拒绝回答

**L9.您上个月获得的加班工资一共有多少？**

[____|____|____|____|____|____|____]元

【访员注意】9999996.加班工资高于百万位数、9999997.不适用、9999998.不知道、9999999.拒绝回答

**L11.请问以下情况在您的工作中经常发生吗？**

| | 一直如此 | 经常 | 有时 | 很少 | 不知道 | 拒绝回答 |
|---|---|---|---|---|---|---|
| **a.做重体力劳动** | 1 | 2 | 3 | 4 | 98 | 99 |
| **b.做重脑力劳动** | 1 | 2 | 3 | 4 | 98 | 99 |
| **c.感觉工作压力大** | 1 | 2 | 3 | 4 | 98 | 99 |

**L12.您平时的工作日程安排是：**

有规律的白班..........................................................................1
有规律的夜班..........................................................................2
轮休制....................................................................................3
工作日程经常变化....................................................................4
其他工作日程..........................................................................5
不知道..................................................................................98
拒绝回答..............................................................................99

**L13.在您的工作中，有关您工作日程的安排，单位或领导一般什么时候给您通知？**

一天之内随时都有可能..............................................................1
提前 1-2 天..............................................................................2
提前 3-6 天..............................................................................3
提前一周到两周........................................................................4
提前两周或更多时间..................................................................5

不知道....................................................................................98
拒绝回答................................................................................99

**L14.过去一个月，您的工作是不是存在电话随叫随到的情况？**

是..........................................................................................1
否..........................................................................................2
不知道....................................................................................98
拒绝回答................................................................................99

**L15.过去一个月，您的工作是不是存在通过微信或电话随时安排工作任务的情况？**

是..........................................................................................1
否..........................................................................................2
不知道....................................................................................98
拒绝回答................................................................................99

**L16.总体而言，感受到如下情况的程度是：**

| | 一直如此 | 经常 | 有时 | 很少 | 从未有过 | 不知道 | 拒绝回答 |
|---|---|---|---|---|---|---|---|
| **a.您的工作妨碍了家庭生活** | 1 | 2 | 3 | 4 | 5 | 98 | 99 |
| **b.您的家庭生活妨碍了工作** | 1 | 2 | 3 | 4 | 5 | 98 | 99 |

**L17.总体而言，您对您当下的工作是否满意？**

非常满意................................................................................1
比较满意................................................................................2
一般......................................................................................3
不太满意................................................................................4
非常不满意............................................................................5
不知道....................................................................................98
拒绝回答................................................................................99

**L18.与去年相比，今年您家的收入状况是：**

增加很多................................................................................1
有增加....................................................................................2
基本没有变化........................................................................3
有减少....................................................................................4
减少很多................................................................................5
不知道....................................................................................98
拒绝回答................................................................................99

**L19.总体而言，过去一年中，您家的收入和支出是什么状况：**

收入多于支出（包括消费和负债还款）...................................1
收支基本平衡........................................................................2
收入难以覆盖支出..................................................................3

不知道........................................................................................98
拒绝回答....................................................................................99

**L20.过去一年中，您是否以提前透支的方式（如信用卡、花呗等）购买生活用品？**

经常使用......................................................................................1
偶尔使用......................................................................................2
从未使用......................................................................................3
不知道........................................................................................98
拒绝回答....................................................................................99

**L21.假设在未来一个月中，您家庭需要一笔 10000 元的支出，在无借贷的情况下您是否有能力支付？**

没有任何问题..............................................................................1
基本上可以..................................................................................2
有些难度......................................................................................3
无力支付......................................................................................4
不知道........................................................................................98
拒绝回答....................................................................................99

**A60a.下列各种情形，哪一种更符合您最近那份非农工作的状况？**

自己是老板（或者是合伙人）.................................................. 1
个体工商户................................................................................ 2
受雇于他人（有固定雇主）...................................................... 3 → **跳问 A60b**
劳务工/劳务派遣人员................................................................ 4 → **跳问 A60b**
零工、散工（无固定雇主的受雇者）...................................... 5 → **跳问 A60c**
在自己家的生意/企业中工作/帮忙，领工资............................ 6 → **跳问 A60b**
在自己家的生意/企业中工作/帮忙，不领工资........................ 7 → **跳问 A60c**
自由职业者................................................................................ 8 → **跳问 A60c**
其他（请注明：________________）...................................... 9 → **跳问 A60b**
不知道........................................................................................ 98
拒绝回答.................................................................................... 99

**【CAPI】当 A58 选 2、5 时回答该题**

**A60a1.请问您雇有的雇员数是**

记录：[____|____|____|____|____]人 → **跳问 A60c**

【访员注意】99996.雇有人数超过五位数、99998.不知道、99999.拒绝回答

**A60b.您最近那份非农工作是否与用人单位或雇主签订了书面劳动合同？**

没有签订劳动合同...................................................................... 1
签有无固定期限劳动合同.......................................................... 2
签有固定期限劳动合同，期限为[____|____|____]月 ............. 3

【访员注意】998.不知道、999.拒绝回答

**A60c.从您第一份非农工作到您最近那份非农工作，您一共工作了多少年？**

记录：[____|____]年

【访员注意】

1.向上取整；

2.98.不知道、99.拒绝回答。

**A60d1.您最近那份非农工作的具体职业名称是：**

[________________________________________]

【访员注意】98.不知道、99.拒绝回答

**A60d2.您最近那份非农工作的具体工作内容是：**

[________________________________________]

[________________________________________]

【访员注意】98.不知道、99.拒绝回答

**A60e.您最近那份非农工作的性质是？**

全职工作........................................................................ 1

非全职工作...................................................................... 2

不知道.......................................................................... 98

拒绝回答........................................................................ 99

**A60f.在您最近那份非农工作中，您的管理活动情况是：**

只管理别人，不受别人管理........................................ 1

既管理别人，又受别人管理........................................ 2

只受别人管理，不管理别人........................................ 3 → **跳问 A60g**

既不管理别人，又不受别人管理.................................. 4 → **跳问 A60g**

不知道.......................................................................... 98

拒绝回答........................................................................ 99

**A60f1.请问您管理的人数是：**

记录：[____|____|____|____|____]人

【访员注意】

1.管理人数指的是直接管理的人数；

2.99996.管理人数超过五位数、99998.不知道、99999.拒绝回答。

**A60g.在您最近那份非农工作中，您在多大程度上能自主决定您工作的具体方式？**

完全自主决定................................................................ 1

能在一定程度上自主...................................................... 2

在很少程度上自主.......................................................... 3

完全不能自主................................................................ 4

不知道.......................................................................... 98

拒绝回答........................................................................ 99

**A60h.在您最近那份非农工作岗位上，是否经常有人希望通过您的工作便利帮他/她办事？**

总是............................................................................. 1

经常............................................................................. 2

有时........................................................................................ 3
很少........................................................................................ 4
从没有...................................................................................... 5
不知道...................................................................................... 98
拒绝回答.................................................................................. 99

**A60i1.您最近那份非农工作的单位或公司的具体名称（全称）是：**

[________________________________________________]

【访员注意】98.不知道、99.拒绝回答

**A60i2.您最近那份非农工作的单位或公司的主要产品或服务是：**

[________________________________________________]

[________________________________________________]

【访员注意】98.不知道、99.拒绝回答

**A60j.您最近那份非农工作的单位或公司的单位类型是：**

党政机关.................................................................................. 1 → **跳问 A60L**
企业........................................................................................ 2
事业单位.................................................................................. 3
社会团体、居/村委会.............................................................. 4
无单位/自雇 ............................................................................ 5 → **跳问 A60m**
军队........................................................................................ 6 → **跳问 A60m**
其他（请注明：______________） ...................................... 7
不知道...................................................................................... 98
拒绝回答.................................................................................. 99

**A60k.您最近那份非农工作的单位或公司所有制性质是：**

国有或国有控股...................................................................... 1
集体所有或集体控股.............................................................. 2
私有/民营或私有/民营控股.................................................... 3
港澳台资或港澳台资控股...................................................... 4
外资所有或外资控股.............................................................. 5
其他（请注明：______________） ...................................... 6
不知道...................................................................................... 98
拒绝回答.................................................................................. 99

**A60L.您最近那份非农工作的单位或公司有多少员工？**

记录：[____|____|____|____|____]人

【访员注意】99996.员工人数超过五位数、99998.不知道、99999.拒绝回答

**A60m.您在最近那份非农工作的工作单位或公司工作了多少年？**

记录：[____|____]年

【访员注意】98.不知道、99.拒绝回答

## 社会保障

**A61.您目前是否参加了以下社会保障项目？**

| | 参加了 | 没有参加 | 不适用 | 不知道 | 拒绝回答 |
|---|---|---|---|---|---|
| **1. 城市基本医疗保险/新型农村合作医疗保险/公费医疗** | 1 | 2 | 7 | 98 | 99 |
| **2. 城市/农村基本养老保险** | 1 | 2 | 7 | 98 | 99 |
| **3. 商业性医疗保险** | 1 | 2 | 7 | 98 | 99 |
| **4. 商业性养老保险** | 1 | 2 | 7 | 98 | 99 |

## 家庭

**A62.您家 2020 年全年家庭总收入是多少？（高位补零）**

[________|________|________|________|________|________|________]

【访员注意】9999996.全年全家总收入高于百万位数、9999997.不适用、9999998.不知道、9999999.拒绝回答

**A64.您家的家庭经济状况在所在地属于哪一档？**

远低于平均水平................................................................ 1
低于平均水平.................................................................... 2
平均水平.......................................................................... 3
高于平均水平.................................................................... 4
远高于平均水平................................................................ 5
不知道.............................................................................. 98
拒绝回答.......................................................................... 99

**A65.您家现拥有几处房产？**

记录：[____|____]处

【访员注意】98.不知道、99.拒绝回答

**A66.您家是否拥有家用小汽车？**

有 ................................................................................... 1
没有.................................................................................. 2
不知道.............................................................................. 98
拒绝回答.......................................................................... 99

**A67.您家目前是否从事下列投资活动？（多选）**

没有任何投资活动............................................................ 1
股票.................................................................................. 2
基金.................................................................................. 3
债券.................................................................................. 4
期货.................................................................................. 5
权证.................................................................................. 6
炒房.................................................................................. 7
外汇投资.......................................................................... 8

其他（请注明：________________）......................................... 9
不知道.................................................................................................. 98
拒绝回答.............................................................................................. 99

**A68.请问您有几个子女（包括继子继女、养子养女在内，包括已去世子女）？**

记录：儿子[____|____]个
　　　女儿[____|____]个

【访员注意】0.没有、99.拒绝回答

**【CAPI】如以上都为零，则直接跳问 A69**

**A68a.请问您有几个（亲生）子女（包括已去世子女）？**

记录：儿子[____|____]个
　　　女儿[____|____]个

【访员注意】0.没有、99.拒绝回答

**A68b.请问您有几个 18 岁以下未成年子女（包括继子继女、养子养女在内）？**

记录：[____|____]个

【访员注意】0.没有、99.拒绝回答

**A69.您目前的婚姻状况是：【出示示卡 6】**

未婚................................................................................................. 1 → **跳问 A89a**
同居................................................................................................. 2
初婚有配偶........................................................................................ 3
再婚有配偶........................................................................................ 4
分居未离婚........................................................................................ 5
离婚................................................................................................. 6 → **跳问 A70**
丧偶................................................................................................. 7 → **跳问 A70**

**A69a.现在您和您的配偶或同居伴侣住在一起吗？**

住在一起............................................................................................ 1
不住在一起，但同在一个城市................................................................. 2
不住在一起，在同一个省的不同城市.......................................................... 3
不住在一起，在不同省........................................................................... 4
不住在一起，在不同国家........................................................................ 5
不知道.............................................................................................. 98
拒绝回答............................................................................................ 99

**A70.您第一次结婚的时间是？**

记录：[____|____|____|____]年

【访员注意】9997.从未结婚、9998.不知道、9999.拒绝回答

**【CAPI】处于离婚或丧偶状态，填答完本题直接跳问 A89a**

**A71a.请问您目前的配偶或同居伴侣是哪一年出生的？**

记录：[____|____|____|____]年

【访员注意】

1.记录公历年；

2.如果被访者以农历、生肖或其他方式报告自己配偶或同居伴侣的出生年，请换算成公历后再记录；

3.9998.不知道、9999.拒绝回答。

**A71b.请问您与目前的配偶是哪一年结婚的？**

记录：[____|____|____|____]年

【访员注意】9997.从未结婚、9998.不知道、9999.拒绝回答

**A72.您配偶或同居伴侣目前的最高教育程度是：**

没有受过任何教育........................................................................ 1
私塾、扫盲班................................................................................ 2
小学.............................................................................................. 3
初中.............................................................................................. 4
职业高中....................................................................................... 5
普通高中....................................................................................... 6
中专.............................................................................................. 7
技校.............................................................................................. 8
大学专科（成人高等教育）........................................................ 9
大学专科（正规高等教育）........................................................ 10
大学本科（成人高等教育）........................................................ 11
大学本科（正规高等教育）........................................................ 12
研究生及以上................................................................................ 13
其他（请注明：________________）...................................... 14
不知道.......................................................................................... 98
拒绝回答....................................................................................... 99

**A73.您配偶或同居伴侣的政治面貌是：**

群众.............................................................................................. 1
共青团员....................................................................................... 2
民主党派....................................................................................... 3
共产党员....................................................................................... 4
不知道.......................................................................................... 98
拒绝回答....................................................................................... 99

**A74.您配偶或同居伴侣目前的户口登记状况是：**

农业户口....................................................................................... 1
非农业户口................................................................................... 2
居民户口（以前是农业户口）.................................................... 3
居民户口（以前是非农业户口）................................................ 4
军籍.............................................................................................. 5
没有户口....................................................................................... 6
其他（请注明：________________）...................................... 7

不知道........................................................................................98
拒绝回答....................................................................................99

**A75a.您配偶或同居伴侣去年（2020 年）全年的总收入是多少？（高位补零）**

[_______|_______|_______|_______|_______|_______|_______]

【访员注意】9999996.配偶/同居伴侣全年总收入高于百万位数、9999997.不适用、9999998.不知道、9999999.拒绝回答

**A75b.您配偶或同居伴侣去年（2020 年）全年的职业/劳动收入是多少？（高位补零）**

[_______|_______|_______|_______|_______|_______|_______]

【访员注意】9999996.配偶/同居伴侣全年职业/劳动收入高于百万位数、9999997.不适用、9999998.不知道、9999999.拒绝回答

**A76.您配偶或同居伴侣上一周的就业状况是？（高位补零）**

未从事任何以获得经济收入为目的的工作..............................1 → **跳问 A77**
带薪休假，学习、临时停工或季节性歇业等..........................2
停薪休假，学习、临时停工或季节性歇业等..........................3
从事了以取得经济收入为目的的工作（包括参军），
一般每周的工作时间为[____|____|____]小时..........................4 → **跳问 A81**
不知道........................................................................................98
拒绝回答....................................................................................99

**A76a.在您配偶或同居伴侣有工作的时候，通常情况下，您配偶或同居伴侣一周的工作时间大概有多少个小时，包括加班时间？**

记录：[____|____|____]小时 → **跳问 A81**

【访员注意】
1.向上取整；
2.998.不知道、999.拒绝回答。

**A77.您配偶或同居伴侣上一周没有工作的原因是什么？**

在校学习......................................................................................1
丧失劳动能力..............................................................................2
毕业后未工作..............................................................................3
因单位原因失去原工作..............................................................4
因个人原因失去原工作..............................................................5
承包土地被征用..........................................................................6
离/退休 ........................................................................................7
料理家务......................................................................................8
其他（请注明：_______________）......................................9
不知道........................................................................................98
拒绝回答....................................................................................99

**A78.到目前为止，您配偶或同居伴侣连续未工作的时间有多少个月？**

记录：[____|____|____]个月

【访员注意】
1.向上取整；
2.997.从未工作过、998.不知道、999.拒绝回答。

**A79.您配偶或同居伴侣在最近三个月内采取过以下哪些方式寻找工作？（多选）**

未找过工作............................................................................ 1
在职业介绍机构登记求职............................................................ 2
委托亲友找工作........................................................................ 3
利用网络及其他媒体求职............................................................ 4
参加招聘会、自己直接上门询问.................................................. 5
为自己经营做准备..................................................................... 6
其他（请注明：________________）.......................................... 7
不知道.................................................................................... 98
拒绝回答................................................................................. 99

**A80.如有适合的工作，您配偶或同居伴侣能否在两周内开始工作？**

能 ......................................................................................... 1
不能....................................................................................... 2
不知道.................................................................................... 98
拒绝回答................................................................................. 99

**A81.您配偶或同居伴侣的工作经历及状况是？**

目前从事非农工作..................................................................... 1
目前务农，曾经有过非农工作..................................................... 2 → **跳问 A89a**
目前务农，没有过非农工作........................................................ 3 → **跳问 A89a**
目前没有工作，而且只务过农..................................................... 4 → **跳问 A89a**
目前没有工作，曾经有过非农工作............................................... 5 → **跳问 A89a**
从未工作过.............................................................................. 6 → **跳问 A89a**
不知道.................................................................................... 98
拒绝回答................................................................................. 99

**A81a.您配偶或同居伴侣目前是否同时兼有多份工作？**

没有....................................................................................... 1 → **跳问 A82**
有，一共兼有[____|____]份工作 ................................................... 2
不知道.................................................................................... 98
拒绝回答................................................................................. 99

【访员注意】98.不知道、99.拒绝回答

**A81b.那您配偶或同居伴侣目前最主要的工作大约占他职业收入的百分之多少？**

记录：[____|____|____]%

【访员注意】0.最主要的工作是务农→ **跳问 A89a**、998.不知道、999.拒绝回答

访员注意：以下 A82 到 A88 的所有问题，如被访者配偶或同居伴侣有多份工作，则指的是他认为最主要的工作。

**A82.下列各种情形，哪一种更符合您配偶或同居伴侣目前的工作的状况？**

自己是老板（或者是合伙人）...................................................... 1
个体工商户.............................................................................. 2
受雇于他人（有固定雇主）......................................................... 3 → **跳问 A83a**
劳务工/劳务派遣人员................................................................. 4 → **跳问 A83a**
零工、散工（无固定雇主的受雇者）............................................ 5 → **跳问 A83b**

在自己家的生意/企业中工作/帮忙，领工资............................ 6 → **跳问 A83a**
在自己家的生意/企业中工作/帮忙，不领工资........................ 7 → **跳问 A83b**
自由职业者.......................................................................... 8 → **跳问 A83b**
其他（请注明：________________）........................................ 9 → **跳问 A83a**
不知道................................................................................ 98
拒绝回答............................................................................ 99

**A82a.请问您配偶或同居伴侣雇有的雇员人数是：**

记录：[____|____|____|____|____]人 → **跳问 A83b**

【访员注意】99996.雇有人数超过五位数、99998.不知道、99999.拒绝回答

**A83a.您配偶或同居伴侣的目前工作是否与用人单位或雇主签订了书面劳动合同？**

没有签订劳动合同................................................................ 1
签有无固定期限劳动合同...................................................... 2
签有固定期限劳动合同，期限为[____|____|____]月.............. 3
不知道................................................................................ 98
拒绝回答............................................................................ 99

**A83b.在您配偶或同居伴侣目前的工作中，他/她的管理活动情况是：**

只管别人，不受别人管理...................................................... 1
既管理别人，又受别人管理.................................................. 2
只受别人管理，不管理别人.................................................. 3 → **跳问 A84a**
既不管理别人，又不受别人管理........................................... 4 → **跳问 A84a**
不知道................................................................................ 98
拒绝回答............................................................................ 99

**A83c.请问他/她管理的人数是：**

记录：[____|____|____|____|____]人

【访员注意】99996.管理人数超过五位数、99998.不知道、99999.拒绝回答

**A84a.您配偶或同居伴侣目前工作的具体职业名称是：**

[__________________________________________________]

【访员注意】98.不知道、99.拒绝回答

**A84b.您配偶或同居伴侣目前的具体工作内容是：**

[__________________________________________________]
[__________________________________________________]

【访员注意】98.不知道、99.拒绝回答

**A85.您配偶或同居伴侣目前工作的性质是？**

全职工作............................................................................ 1
非全职工作.......................................................................... 2
不知道................................................................................ 98
拒绝回答............................................................................ 99

**A86a.您配偶或同居伴侣目前工作的单位或公司的具体名称(全称)是：**

[________________________________________________]

【访员注意】98.不知道、99.拒绝回答

**A86b.您配偶或同居伴侣目前工作的单位或公司的主要产品或服务是：**

[________________________________________________]

[________________________________________________]

【访员注意】98.不知道、99.拒绝回答

**A87.您配偶或同居伴侣目前工作的单位或公司的单位类型是：**

党政机关............................................................................... 1 → **跳问 A89a**
企业....................................................................................... 2
事业单位............................................................................... 3
社会团体、居/村委会........................................................... 4
无单位/自雇（包括个体户）.................................................. 5 → **跳问 A89a**
军队....................................................................................... 6 → **跳问 A89a**
其他（请注明：______________）...................................... 7
不知道................................................................................... 98
拒绝回答............................................................................... 99

**A88.您配偶或同居伴侣目前工作的单位或公司的所有制性质是：**

国有或国有控股................................................................... 1
集体所有或集体控股........................................................... 2
私有/民营或私有/民营控股.................................................. 3
港澳台资或港澳台资控股.................................................... 4
外资所有或外资控股........................................................... 5
其他（请注明：______________）...................................... 6
不知道................................................................................... 98
拒绝回答............................................................................... 99

**A89a.请问您父亲是哪一年出生的？**

记录：[____|____|____|____]年

【访员注意】
1.记录公历年，如果被访者以农历、生肖或其他方式报告自己父亲的出生年，请换算成公历后再记录（可参照示卡 1）；
2.9998.不知道、9999.拒绝回答。

**A89b.您父亲的最高教育程度是：**

没有受过任何教育................................................................ 1
私塾、扫盲班....................................................................... 2
小学....................................................................................... 3
初中....................................................................................... 4
职业高中............................................................................... 5
普通高中............................................................................... 6
中专....................................................................................... 7
技校....................................................................................... 8

大学专科（成人高等教育）........................................ 9
大学专科（正规高等教育）........................................ 10
大学本科（成人高等教育）........................................ 11
大学本科（正规高等教育）........................................ 12
研究生及以上........................................................ 13
其他（请注明：______________）.............................. 14
不知道................................................................ 98
拒绝回答.............................................................. 99

**A89c.您父亲的政治面貌是：**

群众.................................................................... 1
共青团员.............................................................. 2
民主党派.............................................................. 3
共产党员.............................................................. 4
不知道................................................................ 98
拒绝回答.............................................................. 99

**A89d.请问您 14 岁时，您父亲的就业状况是：**

受雇于他人（有固定雇主）........................................ 1
全职务农.............................................................. 2
兼业务农，同时从事一些非农工作............................. 3
劳务工/劳动派遣人员............................................. 4
零工、散工（无固定雇主的受雇者）........................... 5
在自己家的生意或企业中工作/帮忙，领工资.................. 6
在自己家的生意或企业中工作/帮忙，不领工资............... 7
自由职业者........................................................... 8
个体工商户........................................................... 9
自己是老板（或者是合伙人）.................................... 10
离/退休（不在职）.................................................. 11 → **跳问 A90a**
无业（失业/下岗）.................................................. 12 → **跳问 A90a**
丧失劳动力........................................................... 13 → **跳问 A90a**
在上学且没有工作.................................................. 14 → **跳问 A90a**
料理家务.............................................................. 15 → **跳问 A90a**
已去世................................................................. 16 → **跳问 A90a**
其他（请注明：______________）.............................. 17
不知道................................................................ 98
拒绝回答.............................................................. 99

**A89e1.请问您 14 岁时，您父亲的工作单位名称是：**

[________________________________________]

【访员注意】98.不知道、99.拒绝回答

**A89e2.请问您 14 岁时，您父亲工作的具体职业名称是：**

[________________________________________]

【访员注意】98.不知道、99.拒绝回答

**A89e3.请问您 14 岁时，您父亲的具体工作内容是：**

[________________________________________________]

[________________________________________________]

【访员注意】98.不知道、99.拒绝回答

**A89f.您 14 岁时，您父亲如担任了行政职务，他的职务级别是：**

没有担任任何行政职务........................................................ 0
无级别..................................................................................... 1
股级......................................................................................... 2
副科级..................................................................................... 3
正科级..................................................................................... 4
副处级..................................................................................... 5
正处级..................................................................................... 6
副司局级及以上..................................................................... 7
不知道................................................................................... 98
拒绝回答............................................................................... 99

**A89g.您 14 岁时，您父亲工作的单位或公司的单位类型是：**

党政机关................................................................................. 1 → **跳问 A90a**
企业......................................................................................... 2
事业单位................................................................................. 3
社会团体、居/村委会............................................................. 4
无单位/自雇（包括个体户） ................................................ 5 → **跳问 A90a**
军队......................................................................................... 6 → **跳问 A90a**
其他（请注明：_______________） .................................... 7
不知道................................................................................... 98
拒绝回答............................................................................... 99

**A89h.您 14 岁时，您父亲工作的单位或公司所有制性质是：**

国有或国有控股..................................................................... 1
集体所有或集体控股............................................................. 2
私有/民营或私有/民营控股.................................................... 3
港澳台资或港澳台资控股..................................................... 4
外资所有或外资控股............................................................. 5
其他（请注明：_______________） .................................... 6
不知道................................................................................... 98
拒绝回答............................................................................... 99

**A90a.请问您母亲是哪一年出生的？**

记录：[____|____|____|____]年

【访员注意】

1.记录公历年，如果被访者以农历、生肖或其他方式报告自己母亲的出生年，请换算成公历后再记录（可参照示卡 1）；

2.9998.不知道、9999.拒绝回答。

**A90b.您母亲的最高教育程度是：**

没有受过任何教育......1
私塾、扫盲班......2
小学......3
初中......4
职业高中......5
普通高中......6
中专......7
技校......8
大学专科（成人高等教育）......9
大学专科（正规高等教育）......10
大学本科（成人高等教育）......11
大学本科（正规高等教育）......12
研究生及以上......13
其他（请注明：________________）......14
不知道......98
拒绝回答......99

**A90c.您母亲的政治面貌是：**

群众......1
共青团员......2
民主党派......3
共产党员......4
不知道......98
拒绝回答......99

**A90d.<u>请问您 14 岁时</u>，您母亲的就业状况是：**

受雇于他人（有固定雇主）......1
全职务农......2
兼业务农，同时从事一些非农工作......3
劳务工/劳动派遣人员......4
零工、散工（无固定雇主的受雇者）......5
在自己家的生意或企业中工作/帮忙，领工资......6
在自己家的生意或企业中工作/帮忙，不领工资......7
自由职业者......8
个体工商户......9
自己是老板（或者是合伙人）......10
离/退休（不在职）......11 → **跳问 B101**
无业（失业/下岗）......12 → **跳问 B101**
丧失劳动力......13 → **跳问 B101**
在上学且没有工作......14 → **跳问 B101**
料理家务......15 → **跳问 B101**
已去世......16 → **跳问 B101**
其他（请注明：________________）......17
不知道......98
拒绝回答......99

**A90e1.请问您 14 岁时，您母亲的工作单位名称是：**

[________________________________________________]

【访员注意】98.不知道、99.拒绝回答

**A90e2.请问您 14 岁时，您母亲工作的具体职业名称是：**

[________________________________________________]

【访员注意】98.不知道、99.拒绝回答

**A90e3.请问您 14 岁时，您母亲的具体工作内容是：**

[________________________________________________]

[________________________________________________]

【访员注意】98.不知道、99.拒绝回答

**A90f.您 14 岁时，您母亲如担任了行政职务，她的职务级别是：**

没有担任任何行政职务........................................ 0
无级别........................................ 1
股级........................................ 2
副科级........................................ 3
正科级........................................ 4
副处级........................................ 5
正处级........................................ 6
副司局级及以上........................................ 7
不知道........................................ 98
拒绝回答........................................ 99

**A90g.您 14 岁时，您母亲工作的单位或公司的单位类型是：**

党政机关........................................ 1 → **跳问 B101**
企业........................................ 2
事业单位........................................ 3
社会团体、居/村委会........................................ 4
无单位/自雇（包括个体户）........................................ 5 → **跳问 B101**
军队........................................ 6 → **跳问 B101**
其他（请注明：_______________）........................................ 7
不知道........................................ 98
拒绝回答........................................ 99

**A90h.您 14 岁时，您母亲工作的单位或公司所有制性质是：**

国有或国有控股........................................ 1
集体所有或集体控股........................................ 2
私有/民营或私有/民营控股........................................ 3
港澳台资或港澳台资控股........................................ 4
外资所有或外资控股........................................ 5
其他（请注明：_______________）........................................ 6
不知道........................................ 98
拒绝回答........................................ 99

# B 部分

## 择偶观念

**【CAPI】**

**1.A2（性别）用于筛选随机分配的变量；**

**2.生成以下随机变量，每个变量随机产生 3 次，依次用于 B101-B103。**

| | |
|---|---|
| X1 | 若受访者性别为女（A2=2），在[-5, +15]之间生成一个随机整数 n；<br>若受访者性别为男（A2=1），在[-15, +5]之间生成一个随机整数 n；<br>若 n 为负，X1="小您\|n\|岁"；若 n 为正，X1="大您 n 岁"；若 n=0，X1="和您同样大"。 |
| X2 | 随机分配{您收入的 50%（一半），您收入的 60%（六成），您收入的 70%（七成），您收入的 80%（八成），您收入的 90%（九成），和您收入差不多，您收入的 150%（1.5 倍），您收入的 200%（2 倍），您收入的 300%（3 倍）}九项中的一项。 |
| X3 | 随机分配{父母在农村，父母在城市}两项中的一项。 |
| X4 | 随机分配{名下没有房产，名下有房}两项中的一项。 |
| X5 | 随机分配{高中，本科，研究生}三项中的一项。 |
| X6 | 随机分配{有点丑，一般，比较漂亮/比较帅}三项中的一项。如果受访者性别为女（A2=2），第三项显示"比较帅"，如果受访者性别为男（A2=1），第三项显示"比较漂亮"。 |

**同时，存储本问卷中以下基于个人随机生成的变量：**

| |
|---|
| X1_1/X2_1/X3_1/X4_1/X5_1/X6_1/ |
| X1_2/X2_2/X3_2/X4_2/X5_2/X6_2/ |
| X1_3/X2_3/X3_3/X4_3/X5_3/X6_3/ |

**B101.选择配偶时会考虑很多因素，但很多时候不能完全满足，需要作出权衡。如果让您打分，1 分代表非常不理想，7 分代表非常理想，您觉得甲作为结婚的对象有多理想？**

| | 甲 |
|---|---|
| 年龄 | [加载 X1_1] |
| 收入 | [加载 X2_1] |
| 家庭背景 | [加载 X3_1] |
| 房产 | [加载 X4_1] |
| 教育 | [加载 X5_1] |
| 长相 | [加载 X6_1] |

非常不理想　　　　　　　　　　　　　　　　　　　　　　　　非常理想

| 1 | 2 | 3 | 4 | 5 | 6 | 7 |
|---|---|---|---|---|---|---|

**B102.如果让您打分，1 分代表非常不理想，7 分代表非常理想，您觉得乙作为结婚的对象有多理想？**

| | 乙 |
|---|---|
| 年龄 | [加载 X1_2] |
| 收入 | [加载 X2_2] |
| 家庭背景 | [加载 X3_2] |
| 房产 | [加载 X4_2] |
| 教育 | [加载 X5_2] |
| 长相 | [加载 X6_2] |

非常不理想　　　　　　　　　　　　　　　　　　　　　　　　　　　　非常理想

| | | | | | | |
|---|---|---|---|---|---|---|
| 1 | 2 | 3 | 4 | 5 | 6 | 7 |

**B103.如果让您打分，1 分代表非常不理想，7 分代表非常理想，您觉得丙作为结婚的对象有多理想？**

| | 丙 |
|---|---|
| 年龄 | [加载 X1_3] |
| 收入 | [加载 X2_3] |
| 家庭背景 | [加载 X3_3] |
| 房产 | [加载 X4_3] |
| 教育 | [加载 X5_3] |
| 长相 | [加载 X6_3] |

非常不理想　　　　　　　　　　　　　　　　　　　　　　　　　　　　非常理想

| | | | | | | |
|---|---|---|---|---|---|---|
| 1 | 2 | 3 | 4 | 5 | 6 | 7 |

**B2.您觉得婚姻的首要目的是什么？**

个人幸福..........................................................................................1
生养子女..........................................................................................2
经济上互相扶持..............................................................................3
老了有人照顾..................................................................................4
其他..................................................................................................5

## 生育意愿

**【CAPI】系统生成以下 18 种情景条件：**

| 条件 | 收入（5/15/50 万） | 家庭照料（祖辈帮忙/自己带） | 育儿服务（价格低公立/价格低私立/价格高） |
|---|---|---|---|
| 1 | 家庭年收入为 5 万 | 父母或公婆帮忙带孩子 | 家附近有价格低、优质的公立幼儿园和托儿所 |
| 2 | 家庭年收入为 15 万 | 父母或公婆帮忙带孩子 | 家附近有价格低、优质的公立幼儿园和托儿所 |
| 3 | 家庭年收入为 50 万 | 父母或公婆帮忙带孩子 | 家附近有价格低、优质的公立幼儿园和托儿所 |
| 4 | 家庭年收入为 5 万 | 自己带孩子 | 家附近有价格低、优质的公立幼儿园和托儿所 |
| 5 | 家庭年收入为 15 万 | 自己带孩子 | 家附近有价格低、优质的公立幼儿园和托儿所 |
| 6 | 家庭年收入为 50 万 | 自己带孩子 | 家附近有价格低、优质的公立幼儿园和托儿所 |
| 7 | 家庭年收入为 5 万 | 父母或公婆帮忙带孩子 | 家附近有价格低、优质的私立幼儿园和托儿所 |
| 8 | 家庭年收入为 15 万 | 父母或公婆帮忙带孩子 | 家附近有价格低、优质的私立幼儿园和托儿所 |
| 9 | 家庭年收入为 50 万 | 父母或公婆帮忙带孩子 | 家附近有价格低、优质的私立幼儿园和托儿所 |
| 10 | 家庭年收入为 5 万 | 自己带孩子 | 家附近有价格低、优质的私立幼儿园和托儿所 |
| 11 | 家庭年收入为 15 万 | 自己带孩子 | 家附近有价格低、优质的私立幼儿园和托儿所 |
| 12 | 家庭年收入为 50 万 | 自己带孩子 | 家附近有价格低、优质的私立幼儿园和托儿所 |
| 13 | 家庭年收入为 5 万 | 父母或公婆帮忙带孩子 | 家附近只有价格高的公立或私立幼儿园和托儿所 |
| 14 | 家庭年收入为 15 万 | 父母或公婆帮忙带孩子 | 家附近只有价格高的公立或私立幼儿园和托儿所 |
| 15 | 家庭年收入为 50 万 | 父母或公婆帮忙带孩子 | 家附近只有价格高的公立或私立幼儿园和托儿所 |
| 16 | 家庭年收入为 5 万 | 自己带孩子 | 家附近只有价格高的公立或私立幼儿园和托儿所 |
| 17 | 家庭年收入为 15 万 | 自己带孩子 | 家附近只有价格高的公立或私立幼儿园和托儿所 |
| 18 | 家庭年收入为 50 万 | 自己带孩子 | 家附近只有价格高的公立或私立幼儿园和托儿所 |

**【CAPI】随机分配 18 个条件组合中的两项（不放回抽样，两项不一样），生成随机变量 C101_a、C102_a 并存储**
**【CAPI】随机分配{1=“儿子”，2=“女儿”}两项中的一项，生成随机变量 C101_b、C102_b 并存储**

**C101. 32 岁的王女士已经有了一个[加载 C101_b]，在下面的条件下您觉得她应该生二胎吗？请按照 1-5 分进行打分，1 分代表非常不应该，5 分代表非常应该。**

条件[加载 C101_a]

**C102. 32 岁的张女士已经有了一个[加载 C102_b]，在下面的条件下您觉得她应该生二胎吗？请按照 1-5 分进行打分，1 分代表非常不应该，5 分代表非常应该。**

条件[加载 C102_a]

**【CAPI】等比例循环发放 18 个条件组合，生成变量 C103_a**
**【CAPI】随机分配{1=“两个儿子”，2=“两个女儿”，3=“一个儿子和一个女儿”}三项中的一项，生成随机变量 C103_b 并存储**

**C103. 32 岁的李女士已经有了[加载 C103_b]，如果政策条件允许，在下面的条件下您觉得她应该生三胎吗？请按照 1-5 分进行打分，1 分代表非常不应该，5 分代表非常应该。**

条件[加载 C103_a]

## 生育、养育和家庭的社会态度

**C2.您是否同意以下观点？【出示示卡 7】**

| | 非常不同意 | 不同意 | 不同意也不反对 | 同意 | 非常同意 | 不知道 | 拒绝回答 |
|---|---|---|---|---|---|---|---|
| **C201.** 相比于母亲，父亲不应当因为照顾孩子而放缓事业上的进取 | 1 | 2 | 3 | 4 | 5 | 98 | 99 |
| **C202.** 为人母是对女性来说最有成就感的事情之一 | 1 | 2 | 3 | 4 | 5 | 98 | 99 |
| **C203.** 父母应当为子女购买房子 | 1 | 2 | 3 | 4 | 5 | 98 | 99 |
| **C204.** 孩子是家中最重要的人 | 1 | 2 | 3 | 4 | 5 | 98 | 99 |
| **C205.** 需要有自己的房产才可以生孩子 | 1 | 2 | 3 | 4 | 5 | 98 | 99 |
| **C206.** 不管本人想不想生孩子，只要父母想要，我们就应该满足父母的意愿而生孩子 | 1 | 2 | 3 | 4 | 5 | 98 | 99 |

## 疫苗

**V1.目前，您有没有接种新冠疫苗？**

接种了........................................................................................1
没接种........................................................................................2 → **跳问 V5**
不愿回答....................................................................................99 → **跳问 D27**

**V2.您接种第一剂（针）疫苗的时间是？**

记录：[____|____|____|____]年[____|____]月

【访员注意】
1.年份：9998.不知道、9999.拒绝回答；
2.月份：98.不知道、99.拒绝回答。

**V3.您接种疫苗是有人组织的吗？**

是，单位通知/组织........................................................................1
是，社区通知/组织........................................................................2
没有人组织，自行前往接种...........................................................3
不愿回答....................................................................................99

**V4.下列哪种说法最符合您的情况？**

开始不想接种，但最后还是去了...................................................1
开始有些犹豫，但最后还是去了...................................................2
自己原本就想接种...........................................................................3 → **跳问 D27**
不愿回答....................................................................................99 → **跳问 D27**

**V5.您不想接种新冠疫苗的主要原因是？（可多选）**

不符合接种要求...............................................................................1
我国疫情控制得很好，没必要接种...............................................2
其他人都接种了，自己没必要再接种...........................................3
担心接种后，预防效果并不好.......................................................4
担心接种后，对健康产生不良影响...............................................5
担心政府对疫苗管理不力（假疫苗、失效疫苗等）...................6
不知道哪里可以接种.......................................................................7
嫌接种太麻烦...................................................................................8
想接种其他国家的疫苗...................................................................9
其他.................................................................................................10
不愿回答.........................................................................................99

## 疫情

**D27.疫情严重期间，您认为我国政府是否有权力做下列事情？【出示示卡 8】**

| | 当然有权力 | 可能有权力 | 可能没权力 | 当然没权力 | 无法选择 | 拒绝回答 |
|---|---|---|---|---|---|---|
| **a.** 关闭企业或工作场所 | 1 | 2 | 3 | 4 | 98 | 99 |

| | 当然有权力 | 可能有权力 | 可能没权力 | 当然没权力 | 无法选择 | 拒绝回答 |
|---|---|---|---|---|---|---|
| **b.** 要求人们待在家里 | 1 | 2 | 3 | 4 | 98 | 99 |
| **c.** 通过数字设备(如移动电话)监控追踪被感染者 | 1 | 2 | 3 | 4 | 98 | 99 |
| **d.** 要求人们戴口罩 | 1 | 2 | 3 | 4 | 98 | 99 |
| **e.** 禁止公共集会 | 1 | 2 | 3 | 4 | 98 | 99 |

**D29.疫情严重期间，您认为我国政府是否有权力做以下事情？【出示示卡 8】**

| | 当然有权力 | 可能有权力 | 可能没权力 | 当然没权力 | 无法选择 | 拒绝回答 |
|---|---|---|---|---|---|---|
| **a.** 隔离感染者 | 1 | 2 | 3 | 4 | 98 | 99 |
| **b.** 暂时关闭中小学和幼儿园 | 1 | 2 | 3 | 4 | 98 | 99 |
| **c.** 关闭国境 | 1 | 2 | 3 | 4 | 98 | 99 |

**D30.我国应对新冠疫情采取的措施，让您对以下方面的信心产生了什么变化？**

| | 增长了很多 | 增长了一点 | 基本上没变化 | 降低了一点 | 降低了很多 | 无法选择 | 拒绝回答 |
|---|---|---|---|---|---|---|---|
| **a.** 对医疗卫生系统的信心 | 1 | 2 | 3 | 4 | 5 | 98 | 99 |
| **b.** 对政府的信心 | 1 | 2 | 3 | 4 | 5 | 98 | 99 |

**D31.您目前的就业状况与新冠疫情之前相比，下列哪项描述最符合您的情况？**

我在疫情前没有工作，现在也没工作........................................1
我疫情前后的工作没有变化........................................................2
我因为疫情失去了工作，现在有了一份新的工作....................3
我因为疫情失去了工作，现在也没找到新的工作....................4
我在疫情前没有工作，现在有了工作........................................5
我换工作或离职的原因与疫情无关............................................6
无法选择......................................................................................98
拒绝回答......................................................................................99

**D32.您家目前的收入与新冠疫情之前相比有什么变化？**

增长了很多....................................................................................1
增长了一点....................................................................................2
和疫情前一样................................................................................3
下降了一点....................................................................................4
下降了很多....................................................................................5
无法选择......................................................................................98
拒绝回答......................................................................................99

**D33.与新冠疫情之前相比，您目前与家人或朋友见面的频率有什么变化？**

少很多……1
少一点……2
基本一样……3
多一点……4
多很多……5
无法选择……98
拒绝回答……99

**D34.您同不同意以下说法？【出示示卡 15】**

| | 非常同意 | 同意 | 说不上同意不同意 | 不同意 | 非常不同意 | 无法选择 | 拒绝回答 |
|---|---|---|---|---|---|---|---|
| **a.** 多数人变得很胖是因为他们懒 | 1 | 2 | 3 | 4 | 5 | 98 | 99 |
| **b.** 多数人感染新冠是因为他们不小心 | 1 | 2 | 3 | 4 | 5 | 98 | 99 |

# C 部分 主题模块

## EASS

## A 健康状况

**E1.您觉得您目前的身体健康状况是:【出示示卡 9】**

非常好........................................................................................1
很好............................................................................................2
好................................................................................................3
一般............................................................................................4
差................................................................................................5

下面是一些关于您日常活动的问题，您的健康状况是否限制了您参加这些活动？如果是，您受限制的程度如何？

**E2.一般活动，如挪动桌子，使用吸尘器，打保龄球或高尔夫球：**

是的，非常受限........................................................................1
是的，有些受限........................................................................2
不，一点也不受限....................................................................3

**E3.爬几层楼：**

是的，非常受限........................................................................1
是的，有些受限........................................................................2
不，一点也不受限....................................................................3

在过去的四周中，您的健康问题对您的工作或者其他日常活动的影响程度如何？

**E4.由于健康问题，您无法完成预期的工作或日常活动:【出示示卡 10】**

总是............................................................................................1
经常............................................................................................2
有时............................................................................................3
很少............................................................................................4
从来没有....................................................................................5

在过去的四周中，您的任何情绪问题对您的工作或者其他日常活动的影响程度如何（如感到沮丧或焦虑）？

**E6.由于情绪问题，您无法完成预期的工作或日常活动:【出示示卡 10】**

总是............................................................................................1
经常............................................................................................2
有时............................................................................................3
很少............................................................................................4
从来没有....................................................................................5

**E7.由于情绪问题，让您的工作或其他日常活动变得心不在焉:【出示示卡 10】**

总是............................................................................................1
经常............................................................................................2
有时............................................................................................3

很少 ........................................................................................4
从来没有 ................................................................................5

**E8.在过去的四周中，病痛在多大程度上影响您的正常工作（包括家务活和工作）？**

毫无影响 ................................................................................1
稍微有点影响 ........................................................................2
影响一般 ................................................................................3
有比较大的影响 ....................................................................4
有非常大的影响 ....................................................................5

在过去的四周中，您感觉怎么样？请选择一个最接近您真实感受的选项。

**E9.您是否感到心平气和？【出示示卡 10】**

总是 ........................................................................................1
经常 ........................................................................................2
有时 ........................................................................................3
很少 ........................................................................................4
从来没有 ................................................................................5

**E10.您是否充满活力？【出示示卡 10】**

总是 ........................................................................................1
经常 ........................................................................................2
有时 ........................................................................................3
很少 ........................................................................................4
从来没有 ................................................................................5

**E12.在过去的四周中，您的身体健康或情绪问题对您的社会活动（如拜访朋友、亲戚等）的影响程度如何？【出示示卡 10】**

总是 ........................................................................................1
经常 ........................................................................................2
有时 ........................................................................................3
很少 ........................................................................................4
从来没有 ................................................................................5

**E13.您是否患有慢性病或者有长期的健康问题？**

是 ............................................................................................1
否 ............................................................................................2 **→ 跳问 E15a**

**E14.您患有哪些慢性病？请选出所有符合您自身情况的选项。（多选）**

高血压 ....................................................................................1
糖尿病 ....................................................................................2
血脂异常 ................................................................................3
心脏病 ....................................................................................4
中风 ........................................................................................5
癌症 ........................................................................................6
精神障碍 ................................................................................7
肌肉骨骼疾病（如关节炎、腰痛、膝盖问题、关节疼痛、

风湿病）........................................................................................8
呼吸道疾病（如哮喘、肺气肿）............................................9
肾病..............................................................................................10
肝病..............................................................................................11
消化系统疾病（如溃疡）........................................................12
其他（请注明：____________）..........................................13

**E15a.您眼睛近视吗？**

是..................................................................................................1
否..................................................................................................2 → **跳问 E18**

**E15b.您平时戴眼镜吗（包括隐形眼镜）？**

是..................................................................................................1
否..................................................................................................2

# B 健康相关行为

**E18.请问您抽烟吗?**

现在抽..........................................................................................1
以前抽，现在不抽......................................................................2
几乎不抽/从来不抽.....................................................................3

**E19.您喝酒的频率如何?**

每天喝..........................................................................................1
一周几次......................................................................................2
一个月几次..................................................................................3
一年几次甚至更少......................................................................4
我不喝酒......................................................................................5

**E20.您在工作日走多长时间路？（包括您工作和居家生活，从一个地方到另一个地方，以及娱乐、锻炼或休闲而走的路）**

不足 15 分钟................................................................................1
15-29 分钟....................................................................................2
30-59 分钟....................................................................................3
60-89 分钟....................................................................................4
90 分钟及以上..............................................................................5

**E21.您每周从事让您呼吸快过一般情况的体力活动有多少时间？（包括慢跑、骑自行车、锻炼、搬东西、体力劳动、做家务等，但不包括一般走路）**

从来没有......................................................................................1
不足 15 分钟................................................................................2
15-29 分钟....................................................................................3
30-59 分钟....................................................................................4
大约 1 个小时..............................................................................5
大约 2 个小时..............................................................................6
大约 3 个小时..............................................................................7

大约 4 个小时……………………………………………………………8
大约 5 个小时……………………………………………………………9
6 个小时及以上…………………………………………………………10

**E22.您一般工作日有多少时间是坐着的？包括所有坐着的时间（例如坐在桌前，读书，坐着或躺着看电视等）。**

[____|____]小时[____|____]分钟

**E23.您一般工作日的实际睡眠时间是多长？（这不是指所有躺床上的时间，也不包括打盹）**

[____|____]小时[____|____]分钟

**E24. 过去一个月，您如何评价自己的睡眠质量？**

非常好………………………………………………………………………1
比较好………………………………………………………………………2
比较差………………………………………………………………………3
非常差………………………………………………………………………4

**E25.在过去的三年里，您有没有做过任何健康体检？**

有，并且是定期体检……………………………………………………1
有，但是不定期…………………………………………………………2
没有体检过………………………………………………………………3

# C 医疗服务

**E26.在过去的一年中，您就医的频率如何？（这里指的是您自己生病或受伤, 不包括在医院陪伴或探望病人）**

一周数次……………………………………………………………………1
大概一周一次……………………………………………………………2
大概一月一次……………………………………………………………3
一年几次……………………………………………………………………4
大概一年一次……………………………………………………………5
从未…………………………………………………………………………6

**E27.您是否担心下列情况发生在您或者您家人身上？**

**a.当需要医疗服务的时候，不能获得**

非常担心……………………………………………………………………1
有点担心……………………………………………………………………2
不是很担心………………………………………………………………3
完全不担心………………………………………………………………4

**b.当患重病的时候，付不起医药费用**

非常担心……………………………………………………………………1
有点担心……………………………………………………………………2
不是很担心………………………………………………………………3
完全不担心………………………………………………………………4

**E28.在过去的一年里，当您生病或受伤（包括感冒）时，是否有意不去看医生？**

是 ........................................................................................ 1
否 ........................................................................................ 2 → **跳问 E30**
在过去一年里我没有生病或受伤 ........................................ 3 → **跳问 E30**

**E29.为什么？请圈选所有合适的选项（多选）**

等候时间太长了 ........................................................................ 1
费用太高 .................................................................................... 2
附近没有医院或诊所 ................................................................ 3
不知道去哪里就医 .................................................................... 4
就医交通不便 ............................................................................ 5
不喜欢去看医生 ........................................................................ 6
没有时间看医生 ........................................................................ 7
没有必要去看医生 .................................................................... 8
没有医疗保险 ............................................................................ 9
其他(请注明：________) ........................................................ 10
不想感染新冠 ............................................................................ 12

## D 医疗保险/社会保险

**E30.您享有下列哪种形式的医疗保险？**

只有公共医疗保险 .................................................................... 1
公共医疗保险和商业保险 ........................................................ 2
只有商业保险 ............................................................................ 3
没有医疗保险 ............................................................................ 4
不确定 ........................................................................................ 5

## E 传统中医

**E31.在过去的一年里，您是否接受过下列治疗？**

| | 是 | 否 |
|---|---|---|
| **A.针灸** | 1 | 2 |
| **B.艾灸** | 1 | 2 |
| **C.拔火罐** | 1 | 2 |
| **D.中草药治疗** | 1 | 2 |
| **E.指压法或按摩** | 1 | 2 |

## F 社会支持/社会信任

**E32.过去一年，通常是否有人倾听您诉说您个人关心的事情？**

是 ........................................................................................ 1
否 ........................................................................................ 2 → **跳问 E34**
我没有什么关心的事情 ........................................................ 3 → **跳问 E34**

**E33.他们都是哪些人？（多选）**

同住的家庭成员 ........................................................................1
其他亲属(包括不住在一起的亲属) ........................................2
工作的同事 ..............................................................................3
邻居 .........................................................................................4
朋友 .........................................................................................5
医生、照料、治疗等专业人员 ................................................6
其他(请注明：________) .........................................................7

**E34.总的来说，您认为大多数人是可以信任的还是您在与人交往的时候不得不小心谨慎？**

人们几乎总是可以信任的 .......................................................1
人们经常是可以信任的 ...........................................................2
您经常不得不小心谨慎地应付人 ............................................3
您几乎总是小心谨慎地应付人 ................................................4

# G 环境

**E35.在您居住的地方，下面这些问题的严重程度如何？【出示示卡 12】**

| | 非常严重 | 比较严重 | 不太严重 | 一点也不严重 |
|---|---|---|---|---|
| **A.**空气污染 | 1 | 2 | 3 | 4 |
| **B.**水污染 | 1 | 2 | 3 | 4 |
| **C.**噪音污染 | 1 | 2 | 3 | 4 |
| **D.**光照不足 | 1 | 2 | 3 | 4 |

**E36.下面我问的是在您家周围一公里（步行约 15 分钟）范围内的情况，您在多大程度上同意下面的说法？【出示示卡 13】**

| | 完全同意 | 同意 | 既不同意也不反对 | 不同意 | 完全不同意 |
|---|---|---|---|---|---|
| **A.** 我居住的地方适合进行体育锻炼，如慢跑、步行 | 1 | 2 | 3 | 4 | 5 |
| **B.** 在我居住的地方，有很多新鲜的蔬菜和水果可供选择 | 1 | 2 | 3 | 4 | 5 |
| **C.** 在我居住的地方有足够的公共设施（如社区中心、图书馆、公园等） | 1 | 2 | 3 | 4 | 5 |
| **D.** 我居住的地方很安全 | 1 | 2 | 3 | 4 | 5 |
| **E.** 我周围的邻里彼此之间互相关心 | 1 | 2 | 3 | 4 | 5 |
| **F.** 在我有需要的时候，邻居愿意帮助我 | 1 | 2 | 3 | 4 | 5 |

## H 流行病

**E37.去年您是否接种过流感疫苗？**

是......1
否......2

**E38.您有多担心感染上新冠？**

非常担心......1
有些担心......2
不太担心......3
一点也不担心......4

**E39.“如果有人感染了新冠病毒，那是他们自己的过错”，您是否同意？**

同意......1
比较同意......2
比较不同意......3
不同意......4

**E40.“如果我感染了新冠病毒，那是我自己的过错”，您是否同意？**

同意......1
比较同意......2
比较不同意......3
不同意......4

**E41.您认为自己感染新冠的可能性有多大：**

| 极有可能 ⟵ | | | | | | ⟶ 极不可能 |
|---|---|---|---|---|---|---|
| 1 | 2 | 3 | 4 | 5 | 6 | 7 |

## I 家庭护理及需求

**E42.在您同住和不同住的家庭成员中，有没有人因为长期的身心疾病、残疾，或者年老体弱，而需要照顾？**

是......1
否......2 → **跳问 E44**

**E43.您是否是其中一位的主要照料者？**

是......1
否......2

## J 老龄化的顾虑

**E44.**下面我想问一下您对于年老的看法，请问您多大程度上同意下面的说法。【出示示卡 **13**】

| | 完全同意 | 同意 | 既不同意也不反对 | 不同意 | 完全不同意 |
|---|---|---|---|---|---|
| **A.** 我担心当我年老时生活不能够自理 | 1 | 2 | 3 | 4 | 5 |
| **B.** 我担心当我年老时不得不让别人替我拿主意 | 1 | 2 | 3 | 4 | 5 |
| **C.** 在经济上依赖别人是我对年老最大的担心之一 | 1 | 2 | 3 | 4 | 5 |

**E45.**健康对您以下各方面的重要性如何？

| | 非常重要 | 重要 | 有点重要 | 不重要 |
|---|---|---|---|---|
| **A.**免于身体的痛苦 | 1 | 2 | 3 | 4 |
| **B.**内心平和，精神镇定 | 1 | 2 | 3 | 4 |
| **C.**履行家庭角色和责任 | 1 | 2 | 3 | 4 |
| **D.**履行工作、社会责任和角色 | 1 | 2 | 3 | 4 |

**E46.**当您阅读医嘱、药物说明书或其他书面材料时，您需要有人帮助您的频繁程度如何？【出示示卡 **14**】

从不........................................................................................1
很少........................................................................................2
有时........................................................................................3
经常........................................................................................4
总是........................................................................................5

**E47.**当您填写个人档案、病史、知情同意书等医疗表格时，您是否有信心能填好?

完全有信心..............................................................................1
相当有信心..............................................................................2
比较有信心..............................................................................3
有一点信心..............................................................................4
完全没信心..............................................................................5

**E48.在没有他人帮忙的情况下，您是否经常遇到下列情况？【出示示卡 14】**

| | 从不 | 很少 | 有时 | 经常 | 总是 |
|---|---|---|---|---|---|
| **A.** 因为听不懂医护人员的说明，而无法清楚知道自己的健康状况？ | 1 | 2 | 3 | 4 | 5 |
| **B.** 因为不知道如何向医护人员问问题，而无法清楚知道自己的健康状况？ | 1 | 2 | 3 | 4 | 5 |
| **C.** 因为看不懂药品说明书或医嘱，而无法知道如何照顾自己的健康？ | 1 | 2 | 3 | 4 | 5 |

**E49a.您平常几点睡觉？___时___分（请用 24 小时制，如晚上 12 点半，记为 00 时 30 分）**

**E49b.您平常几点起床？___时___分（请用 24 小时制，如早上 7 点半，记为 7 时 30 分）**

**E50.您同意以下说法吗？**

| | 完全同意 | 同意 | 不同意 | 完全不同意 |
|---|---|---|---|---|
| **A.** 老年人从政府得到的东西比他们应得的多 | 1 | 2 | 3 | 4 |
| **B.** 老年人是社会的负担 | 1 | 2 | 3 | 4 |
| **C.** 老年人有太大的政治影响力 | 1 | 2 | 3 | 4 |

**E51.总的来说，您认为您配偶的健康状况是:【出示示卡 9】**

非常好......1
很好......2
好......3
一般......4
差......5

**【CAPI】当 A69 选 1、6、7 时，该题不出现**

# ISSP

**D1.总的来说，您觉得您的生活是否幸福？**

完全幸福......1
非常幸福......2
比较幸福......3
说不上幸福不幸福......4
比较不幸福......5
非常不幸福......6
完全不幸福......7
不知道......98
拒绝回答......99

**D2.总的来说，您对中国的医疗卫生有多大的信心？**

完全有信心......1
有很大信心......2
有一些信心......3

几乎没信心……4
完全没有信心……5
无法选择……98
拒绝回答……99

**D3.比起低收入的人，高收入的人能够负担得起更好的医疗保健，您认为这公平吗？**

非常公平……1
比较公平……2
说不上公平不公平……3
比较不公平……4
非常不公平……5
无法选择……98
拒绝回答……99

**D4.您同不同意以下说法？【出示示卡 15】**

| | 非常同意 | 同意 | 说不上同意不同意 | 不同意 | 非常不同意 | 无法选择 | 拒绝回答 |
|---|---|---|---|---|---|---|---|
| **a.** 人们在使用医疗卫生服务时，往往超出了必要 | 1 | 2 | 3 | 4 | 5 | 98 | 99 |
| **b.** 政府应当只提供基本的医疗卫生保健服务 | 1 | 2 | 3 | 4 | 5 | 98 | 99 |
| **c.** 总的来说，中国的医疗卫生系统没有效率 | 1 | 2 | 3 | 4 | 5 | 98 | 99 |

**D5.您是否愿意交更高的税，来提高中国的全民医疗卫生水平？**

很愿意……1
比较愿意……2
说不上愿意不愿意……3
比较不愿意……4
很不愿意……5
无法选择……98
拒绝回答……99

**D6.您同意以下说法吗？【出示示卡 15】**

| | 非常同意 | 同意 | 说不上同意不同意 | 不同意 | 非常不同意 | 无法选择 | 拒绝回答 |
|---|---|---|---|---|---|---|---|
| **a.** 人们应当享受公费医疗，即便他们没有中国国籍 | 1 | 2 | 3 | 4 | 5 | 98 | 99 |

| | 非常同意 | 同意 | 说不上同意不同意 | 不同意 | 非常不同意 | 无法选择 | 拒绝回答 |
|---|---|---|---|---|---|---|---|
| **b.** 人们应当享受公费医疗，即便他们行为方式损害自身健康（比如抽烟，吸毒） | 1 | 2 | 3 | 4 | 5 | 98 | 99 |

**D7.** 您认为在我国以下各类人群相比较，获得医疗卫生服务的难易程度怎样？【出示示卡 **16**】

| | 容易很多 | 容易一些 | 差不多一样 | 困难一些 | 困难很多 | 无法选择 | 拒绝回答 |
|---|---|---|---|---|---|---|---|
| **a.** 富人比穷人 | 1 | 2 | 3 | 4 | 5 | 98 | 99 |
| **b.** 老年人比年轻人 | 1 | 2 | 3 | 4 | 5 | 98 | 99 |
| **c.** 女性比男性 | 1 | 2 | 3 | 4 | 5 | 98 | 99 |
| **d.** 中国人比外国人 | 1 | 2 | 3 | 4 | 5 | 98 | 99 |

**D8.**有多种原因会导致严重的健康问题。您同不同意以下说法？【出示示卡 **15**】

| | 非常同意 | 同意 | 说不上同意不同意 | 不同意 | 非常不同意 | 无法选择 | 拒绝回答 |
|---|---|---|---|---|---|---|---|
| **a.** 人们出现严重的健康问题是因为他们的行为方式损害自身健康（例如，抽烟，吸毒） | 1 | 2 | 3 | 4 | 5 | 98 | 99 |
| **b.** 人们出现严重的健康问题是因为他们的工作或居住环境 | 1 | 2 | 3 | 4 | 5 | 98 | 99 |
| **c.** 人们出现严重的健康问题是因为遗传 | 1 | 2 | 3 | 4 | 5 | 98 | 99 |
| **d.** 人们出现严重的健康问题是因为他们穷 | 1 | 2 | 3 | 4 | 5 | 98 | 99 |

**D9.**您同意"中医比西医更有效"的说法吗？【出示示卡 **15**】

非常同意......................................................................................1
同意..............................................................................................2
说不上同意不同意........................................................................3
不同意..........................................................................................4

非常不同意……5
无法选择……98
拒绝回答……99

**D10.您同不同意下面这些对于中国医生的说法？【出示示卡 15】**

| | 非常同意 | 同意 | 说不上同意不同意 | 不同意 | 非常不同意 | 无法选择 | 拒绝回答 |
|---|---|---|---|---|---|---|---|
| **a.** 总的来说，医生还是可信的 | 1 | 2 | 3 | 4 | 5 | 98 | 99 |
| **b.** 医生的医术没有他们应该有的那样好 | 1 | 2 | 3 | 4 | 5 | 98 | 99 |
| **c.** 比起关心病人，医生更关心自己的收入 | 1 | 2 | 3 | 4 | 5 | 98 | 99 |

**D11.过去 12 个月，您通过各种形式上网为自己或他人搜索关于健康或医疗方面信息的频繁程度如何？**

我从不上网……0
每天几次……1
每天一次……2
每周几次……3
每月几次……4
一年几次……5
几乎没有……6
无法选择……98
拒绝回答……99

**D12.过去 12 个月，您是否经常上网搜索以下方面的资料？【出示示卡 2】**

| | 从不 | 很少 | 有时 | 经常 | 非常频繁 | 无法选择 | 拒绝回答 |
|---|---|---|---|---|---|---|---|
| **a.** 关于健康生活方式的信息 | 1 | 2 | 3 | 4 | 5 | 98 | 99 |
| **b.** 与焦虑、压力相关或类似的信息 | 1 | 2 | 3 | 4 | 5 | 98 | 99 |
| **c.** 关于接种疫苗的信息 | 1 | 2 | 3 | 4 | 5 | 98 | 99 |

**D13.您同意以下说法吗？【出示示卡 15】**

| | 非常同意 | 同意 | 说不上同意不同意 | 不同意 | 非常不同意 | 无法选择 | 拒绝回答 |
|---|---|---|---|---|---|---|---|
| **a.** 过去 12 个月，互联网上的信息对我的健康行为产生了积极的影响 | 1 | 2 | 3 | 4 | 5 | 98 | 99 |
| **b.** 过去 12 个月，互联网上的信息帮助我理解医生告诉我的事情 | 1 | 2 | 3 | 4 | 5 | 98 | 99 |

**D14.您同意以下说法吗？【出示示卡 15】**

| | 非常同意 | 同意 | 说不上同意不同意 | 不同意 | 非常不同意 | 无法选择 | 拒绝回答 |
|---|---|---|---|---|---|---|---|
| **a.** 互联网可以有助于人们判断自己是否需要去看医生 | 1 | 2 | 3 | 4 | 5 | 98 | 99 |
| **b.** 互联网有助于确认医生是否给了人们适当的建议 | 1 | 2 | 3 | 4 | 5 | 98 | 99 |
| **c.** 网上的健康信息不容易区分是否可靠 | 1 | 2 | 3 | 4 | 5 | 98 | 99 |

**D15.您同意以下关于疫苗的说法吗？【出示示卡 15】**

| | 非常同意 | 同意 | 说不上同意不同意 | 不同意 | 非常不同意 | 无法选择 | 拒绝回答 |
|---|---|---|---|---|---|---|---|
| **a.** 总的来说，接种疫苗坏处多过好处 | 1 | 2 | 3 | 4 | 5 | 98 | 99 |
| **b.** 通过得病获得免疫力好过接种疫苗 | 1 | 2 | 3 | 4 | 5 | 98 | 99 |

**D16.在过去的四个星期中，您是否经常遇到以下问题？【出示示卡 2】**

| | 从不 | 很少 | 有时 | 经常 | 非常频繁 | 无法选择 | 拒绝回答 |
|---|---|---|---|---|---|---|---|
| **b.** 您是否有过身体疼痛的情况？ | 1 | 2 | 3 | 4 | 5 | 98 | 99 |
| **c.** 您是否感到过不开心或是沮丧？ | 1 | 2 | 3 | 4 | 5 | 98 | 99 |
| **d.** 您是否对自己失去过信心？ | 1 | 2 | 3 | 4 | 5 | 98 | 99 |
| **e.** 您是否感到无法克服遇到的困难？ | 1 | 2 | 3 | 4 | 5 | 98 | 99 |

**D17.在过去的 12 个月里，您是否经常去看医生？【出示示卡 2】**

| | 从不 | 很少 | 有时 | 经常 | 非常频繁 | 无法选择 | 拒绝回答 |
|---|---|---|---|---|---|---|---|
| **a.西医** | 1 | 2 | 3 | 4 | 5 | 98 | 99 |
| **b.中医** | 1 | 2 | 3 | 4 | 5 | 98 | 99 |

**D18.在过去的 12 个月里，您是否由于以下原因没能得到必要的医疗？**

| | 是 | 否 | 不需要医疗 | 拒绝回答 |
|---|---|---|---|---|
| **a.您付不起钱** | 1 | 2 | 0 | 99 |
| **b.您无法从工作中请假或是因其他事情脱不开身** | 1 | 2 | 0 | 99 |
| **c.需要等太久** | 1 | 2 | 0 | 99 |

**D19.如果您得了重病，有多大的可能性可以得到国内最好的治疗？**

肯定会得到........................................................................1
可能会得到........................................................................2
可能会也可能不会...............................................................3
可能得不到........................................................................4
肯定得不到........................................................................5
无法选择...........................................................................98
拒绝回答...........................................................................99

**D20.总的来说，您对中国的医疗卫生系统满意吗？**

完全满意............................................................................1
很满意...............................................................................2
比较满意............................................................................3
说不上满意不满意...............................................................4
比较不满意........................................................................5
很不满意............................................................................6
完全不满意........................................................................7
无法选择...........................................................................98
拒绝回答...........................................................................99

**D21.您对接受过的下列治疗满意吗？【出示示卡 17】**

| | 完全满意 | 非常满意 | 比较满意 | 说不上满意不满意 | 比较不满意 | 非常不满意 | 完全不满意 | 不适用 | 无法选择 | 拒绝回答 |
|---|---|---|---|---|---|---|---|---|---|---|
| **a.** 您上一次看西医 | 1 | 2 | 3 | 4 | 5 | 6 | 7 | 97 | 98 | 99 |
| **b.** 您上一次看中医 | 1 | 2 | 3 | 4 | 5 | 6 | 7 | 97 | 98 | 99 |

**D22.您现在抽烟吗？如果抽的话一天几支？**

从来没抽过........................................................................1
现在不抽但以前抽过..........................................................2
每天抽 1-5 支.......................................................................3
每天抽 6-10 支.....................................................................4
每天抽 11-20 支...................................................................5
每天抽 21-40 支...................................................................6
每天抽烟超过 40 支............................................................7
无法选择..............................................................................98
拒绝回答..............................................................................99

**D23.您是否经常做下面这些事？**

| | 从不 | 每月 1 次或更少 | 每月几次 | 每星期几次 | 每天 | 无法选择 | 拒绝回答 |
|---|---|---|---|---|---|---|---|
| **a.** 在一天之内喝酒 **4** 个单位或更多【出示示卡 **18**】 | 1 | 2 | 3 | 4 | 5 | 98 | 99 |
| **b.** 进行至少 **20** 分钟能让您出汗或呼吸加快的身体锻炼 | 1 | 2 | 3 | 4 | 5 | 98 | 99 |
| **c.** 吃新鲜水果或蔬菜 | 1 | 2 | 3 | 4 | 5 | 98 | 99 |

**D24.总的来说，您认为您的健康状况如何（这里说的健康包括生理和心理健康）？【出示示卡 9】**

非常好..................................................................................1
很好......................................................................................2
好..........................................................................................3
一般......................................................................................4
差..........................................................................................5
无法选择..............................................................................98
拒绝回答..............................................................................99

**D25.请问您是否长期生病、或者有慢性病或者有残疾？**

是..........................................................................................1
否..........................................................................................2
拒绝回答..............................................................................99

**D28.请问您目前享有哪些医疗保险？**

没有医疗保险....................................................................1
公费医疗/基本医疗保险（A）..............................................2
个人购买的商业医疗保险（B）............................................3
单位或其他组织提供的附加医疗保险（C）..........................4
公费医疗/基本医疗保险和个人购买的商业医疗保险（A+B）…..................................................................5
公费医疗/基本医疗保险和单位或其他组织提供的附加医疗保险（A+C）..........................................................6
个人购买的商业医疗保险和单位或其他组织提供的附加医疗保险（B+C）......................................................7
公费医疗/基本医疗保险、个人购买的商业医疗保险和单位或其他组织提供的附加医疗保险（A+B+C）........................8
其他类型的医疗保险（请注明：_______）..........................9
不知道...............................................................................98
拒绝回答...........................................................................99

# 主观幸福感

**D35.您对以下观点的同意程度如何？【出示示卡 19】**

| | 非常不同意 | 不同意 | 有点不同意 | 有点同意 | 同意 | 非常同意 | 不知道 | 拒绝回答 |
|---|---|---|---|---|---|---|---|---|
| **1.** 社会给人们提供的出路会越来越多 | 1 | 2 | 3 | 4 | 5 | 6 | 98 | 99 |
| **2.** 随着年龄增长，我从生活中悟出了许多道理，这使我变得更坚强、更有能力 | 1 | 2 | 3 | 4 | 5 | 6 | 98 | 99 |
| **3.** 我设立的生活目标多数能够给我鼓劲，而不是泄气 | 1 | 2 | 3 | 4 | 5 | 6 | 98 | 99 |
| **4.** 我经常感到自己只是在混日子 | 1 | 2 | 3 | 4 | 5 | 6 | 98 | 99 |
| **5.** 我不清楚自己一生所做的事情有什么意义 | 1 | 2 | 3 | 4 | 5 | 6 | 98 | 99 |
| **6.** 我经常感到自己身体某些部位特别不舒服 | 1 | 2 | 3 | 4 | 5 | 6 | 98 | 99 |
| **7.** 与周围的人相比，我很知足 | 1 | 2 | 3 | 4 | 5 | 6 | 98 | 99 |
| **8.** 我对家里的收入感到满意 | 1 | 2 | 3 | 4 | 5 | 6 | 98 | 99 |
| **9.** 我常因一些小事而烦恼 | 1 | 2 | 3 | 4 | 5 | 6 | 98 | 99 |
| **10.** 我很为自己的健康状况感到苦恼 | 1 | 2 | 3 | 4 | 5 | 6 | 98 | 99 |

| | 非常不同意 | 不同意 | 有点不同意 | 有点同意 | 同意 | 非常同意 | 不知道 | 拒绝回答 |
|---|---|---|---|---|---|---|---|---|
| **11. 我常常感到自己很难与他人建立友谊** | 1 | 2 | 3 | 4 | 5 | 6 | 98 | 99 |
| **12. 我比较喜欢自己的个性** | 1 | 2 | 3 | 4 | 5 | 6 | 98 | 99 |
| **13. 我感到似乎大多数人都比我朋友多** | 1 | 2 | 3 | 4 | 5 | 6 | 98 | 99 |
| **14. 和家人在一起，我感到特别愉快** | 1 | 2 | 3 | 4 | 5 | 6 | 98 | 99 |
| **15. 我的运气比别人差** | 1 | 2 | 3 | 4 | 5 | 6 | 98 | 99 |
| **16. 我对社会的发展感到很有信心** | 1 | 2 | 3 | 4 | 5 | 6 | 98 | 99 |
| **17. 与周围人相比，我感到自己挺吃亏** | 1 | 2 | 3 | 4 | 5 | 6 | 98 | 99 |
| **18. 碰到不开心的事情时，很长时间我都打不起精神来** | 1 | 2 | 3 | 4 | 5 | 6 | 98 | 99 |
| **19. 我感到高兴的是，这些年自己的看法变得越来越成熟** | 1 | 2 | 3 | 4 | 5 | 6 | 98 | 99 |
| **20. 我有时感到很难与家人（包括父母、爱人、孩子等）沟通** | 1 | 2 | 3 | 4 | 5 | 6 | 98 | 99 |
| **21. 我对我周围的自然环境感到满意** | 1 | 2 | 3 | 4 | 5 | 6 | 98 | 99 |

**D36.请给您目前的幸福感评分（最高 10 分，最低 0 分）**

记录：[____|____]分

【访员注意】98.不知道、99.拒绝回答

**D37.在幸福感评分中您认为几分以上是幸福的（最高 10 分，最低 0 分）**

记录：[____|____]分

【访员注意】98.不知道、99.拒绝回答

## 环境模块一

**H1.根据您自己的判断，整体上看，您觉得中国目前面临的环境问题是否严重？**

非常严重 ........................................ 5
比较严重 ........................................ 4
既严重也不严重 .................................. 3
不太严重 ........................................ 2
根本不严重 ...................................... 1
无法选择 ........................................ 98

**H2.以下是各种类型的环境问题，您觉得它们在您居住地区的严重程度是怎样的？【出示示卡 20】**

| 环境问题类型 | 很严重 | 比较严重 | 一般 | 不太严重 | 不严重 | 没有该问题 | 无法回答 |
|---|---|---|---|---|---|---|---|
| **1.** 空气污染 | 5 | 4 | 3 | 2 | 1 | 7 | 98 |
| **2.** 水污染 | 5 | 4 | 3 | 2 | 1 | 7 | 98 |
| **3.** 土壤污染 | 5 | 4 | 3 | 2 | 1 | 7 | 98 |
| **4.** 噪声污染 | 5 | 4 | 3 | 2 | 1 | 7 | 98 |
| **5.** 工业垃圾污染 | 5 | 4 | 3 | 2 | 1 | 7 | 98 |
| **6.** 生活垃圾污染 | 5 | 4 | 3 | 2 | 1 | 7 | 98 |
| **7.** 建筑装修污染 | 5 | 4 | 3 | 2 | 1 | 7 | 98 |
| **8.** 森林植被破坏 | 5 | 4 | 3 | 2 | 1 | 7 | 98 |
| **9.** 生物多样性破坏（如野生物种减少） | 5 | 4 | 3 | 2 | 1 | 7 | 98 |
| **10.** 绿地不足 | 5 | 4 | 3 | 2 | 1 | 7 | 98 |
| **11.** 淡水资源短缺 | 5 | 4 | 3 | 2 | 1 | 7 | 98 |
| **12.** 土地荒漠化 | 5 | 4 | 3 | 2 | 1 | 7 | 98 |
| **13.** 水土流失 | 5 | 4 | 3 | 2 | 1 | 7 | 98 |
| **14.** 资源浪费（如水电浪费、废物循环利用不足） | 5 | 4 | 3 | 2 | 1 | 7 | 98 |
| **15.** 天气极端反常（例如气温超常和雨水失调） | 5 | 4 | 3 | 2 | 1 | 7 | 98 |
| **16.** 食品安全 | 5 | 4 | 3 | 2 | 1 | 7 | 98 |

**H3.整体上看，您对造成上述环境问题的原因有多少了解？**

根本不了解 ........................................ 1
不太了解 ........................................ 2
说不上了解不了解 ........................................ 3
比较了解 ........................................ 4
非常了解 ........................................ 5
无法选择 ........................................ 98

**H4.整体上看，您对解决上述各种环境问题的办法有多少了解？**

根本不了解 ........................................ 1
不太了解 ........................................ 2
说不上了解不了解 ........................................ 3
比较了解 ........................................ 4

非常了解 ........................................................................................5
无法选择 ......................................................................................98

**H5.为了解决您和您家庭遭遇的环境问题，您和家人采取任何行动了吗？**

采取了行动 ....................................................................................1
没有采取行动 ..............................................................................2 → **跳问 H6**
试图采取行动，但不知道怎么办.........................................................3 → **跳问 H6**
没有遭遇什么环境问题...................................................................4 → **跳问 H6**

**H5a.如果您和家人采取了行动，主要是哪些类型？（可多选）**

向责任单位或个人直接交涉.........................................................1
向街道、居委会反映.....................................................................2
向工作单位反映...........................................................................3
向居住地政府投诉........................................................................4
通过自媒体和互联网曝光..............................................................5
向正式新闻单位投诉....................................................................6
通过民间环保团体反映..................................................................7
向上级政府投诉...........................................................................8
通过司法渠道解决........................................................................9
组织起来集体维权......................................................................10
公开聚集上访、游行...................................................................12
其他行动（请注明：__________） ..........................................13

**H6.总体看来，您认为您居住地区的环境质量比 5 年前的情况是？**

没有改善........................................................................................1
改善了一些....................................................................................2
不清楚/说不好 ..............................................................................3
改善较多........................................................................................4
有很大改善....................................................................................5
无法选择 ......................................................................................98

**H7.总体看来，您认为您居住地区的环境质量比 10 年前的情况是？**

没有改善........................................................................................1
改善了一些....................................................................................2
不清楚/说不好 ..............................................................................3
改善较多........................................................................................4
有很大改善....................................................................................5
无法选择 ......................................................................................98

**H8.在解决您居住地区环境问题方面，您认为近五年来，地方政府做得怎么样？**

片面注重经济发展，忽视了环境保护工作.......................................1
重视不够，环保投入不足...............................................................2
虽尽了努力，但效果不佳...............................................................3
尽了很大努力，有一定成效.............................................................4
取得了很大的成绩.........................................................................5
无法选择......................................................................................98

**H9.就企业、政府、公民团体和公民个人而言，您认为哪一方最需要对缓解中国面临的环境问题负责任？**

企业 ........................................ 1
政府 ........................................ 2
公民团体 ........................................ 3
公民个人 ........................................ 4
无法选择 ........................................ 98

**H10.您对以下各种事项或者知识点的了解程度是？【出示示卡 21】**

| | 不了解 | 了解一些 | 了解较多 | 很了解 | 无法选择 |
|---|---|---|---|---|---|
| **1.生态文明** | 0 | 1 | 2 | 3 | 98 |
| **2.生态补偿** | 0 | 1 | 2 | 3 | 98 |
| **3.生态保护红线** | 0 | 1 | 2 | 3 | 98 |
| **4.生态文明体制改革** | 0 | 1 | 2 | 3 | 98 |
| **5.国家生态文明试验区** | 0 | 1 | 2 | 3 | 98 |
| **6.生态文明建设目标评价考核** | 0 | 1 | 2 | 3 | 98 |
| **7.国土主体功能区** | 0 | 1 | 2 | 3 | 98 |
| **8.循环经济** | 0 | 1 | 2 | 3 | 98 |
| **9.环保督察巡视** | 0 | 1 | 2 | 3 | 98 |
| **10.大气污染防治行动计划** | 0 | 1 | 2 | 3 | 98 |
| **11.水污染防治行动计划** | 0 | 1 | 2 | 3 | 98 |
| **12.土壤污染防治行动计划** | 0 | 1 | 2 | 3 | 98 |
| **13.环境保护公众参与办法** | 0 | 1 | 2 | 3 | 98 |
| **14.党政领导干部生态环境损害责任追究** | 0 | 1 | 2 | 3 | 98 |

**H11.在解决中国国内环境问题方面，您认为近五年来，中央政府做得怎么样？**

片面注重经济发展，忽视了环境保护工作 ........................................ 1
重视不够，环保投入不足 ........................................ 2
虽尽了努力，但效果不佳 ........................................ 3
尽了很大努力，有一定成效 ........................................ 4
取得了很大的成绩 ........................................ 5
无法选择 ........................................ 98

**H12.我们想了解一下您对人类社会与环境关系的一般看法。请问您对下列说法的同意程度如何？【出示示卡 4】**

| | 完全不同意 | 比较不同意 | 无所谓同意不同意 | 比较同意 | 完全同意 | 无法选择 |
|---|---|---|---|---|---|---|
| 1. 目前的人口总量正在接近地球能够承受的极限 | 1 | 2 | 3 | 4 | 5 | 98 |
| 2. 人是最重要的，可以为了满足自身的需要而改变自然环境 | 1 | 2 | 3 | 4 | 5 | 98 |

| | | | | | | |
|---|---|---|---|---|---|---|
| **3.** 人类对于自然的破坏常常导致灾难性后果 | 1 | 2 | 3 | 4 | 5 | 98 |
| **4.** 由于人类的智慧，地球环境状况的改善是完全可能的 | 1 | 2 | 3 | 4 | 5 | 98 |
| **5.** 目前人类正在滥用和破坏环境 | 1 | 2 | 3 | 4 | 5 | 98 |
| **6.** 只要我们知道如何开发，地球上的自然资源是很充足的 | 1 | 2 | 3 | 4 | 5 | 98 |
| **7.** 动植物与人类有着一样的生存权 | 1 | 2 | 3 | 4 | 5 | 98 |
| **8.** 自然界的自我平衡能力足够强，完全可以应付现代工业社会的冲击 | 1 | 2 | 3 | 4 | 5 | 98 |
| **9.** 尽管人类有着特殊能力，但是仍然受自然规律的支配 | 1 | 2 | 3 | 4 | 5 | 98 |
| **10.** 所谓人类正在面临"环境危机"，是一种过分夸大的说法 | 1 | 2 | 3 | 4 | 5 | 98 |
| **11.** 地球就像宇宙飞船，只有很有限的空间和资源 | 1 | 2 | 3 | 4 | 5 | 98 |
| **12.** 人类生来就是主人，是要统治自然界的其他部分的 | 1 | 2 | 3 | 4 | 5 | 98 |
| **13.** 自然界的平衡是很脆弱的，很容易被打乱 | 1 | 2 | 3 | 4 | 5 | 98 |
| **14.** 人类终将知道更多的自然规律，从而有能力控制自然 | 1 | 2 | 3 | 4 | 5 | 98 |
| **15.** 如果一切按照目前的样子继续，我们很快将遭受严重的环境灾难 | 1 | 2 | 3 | 4 | 5 | 98 |

**H13.**在您生活的社区/村庄中是否存在以下现象？如果存在，您认为这些现象在多大程度上与周边居住环境质量有关？（注：如果第一组提问选择“没有”跳答下一题，如果选择“有”则需回答第二组提问）

第一组提问：

| | 是否存在？ | |
|---|---|---|
| | 没有 | 有 |
| **1.**疾病 | 0 | 1 |
| **2.**精神压抑（如感到恐惧、烦躁） | 0 | 1 |
| **3.**生活质量下降 | 0 | 1 |
| **4.**产业贬值（如房屋等财产贬值） | 0 | 1 |
| **5.**人口逐年外迁/流失 | 0 | 1 |
| **6.**社区萧条 | 0 | 1 |
| **7.**对地方政府信任感下降 | 0 | 1 |
| **8.**民众上访/抗议 | 0 | 1 |

**第二组提问：【出示示卡 22】**

| | 与居住环境质量是否有关？ | | | | |
|---|---|---|---|---|---|
| | 密切相关 | 有关 | 不清楚 | 关系不大 | 没关系 |
| **1.疾病** | 5 | 4 | 3 | 2 | 1 |
| **2.精神压抑（如感到恐惧、烦躁）** | 5 | 4 | 3 | 2 | 1 |
| **3.生活质量下降** | 5 | 4 | 3 | 2 | 1 |
| **4.产业贬值（如房屋等财产贬值）** | 5 | 4 | 3 | 2 | 1 |
| **5.人口逐年外迁/流失** | 5 | 4 | 3 | 2 | 1 |
| **6.社区萧条** | 5 | 4 | 3 | 2 | 1 |
| **7.对地方政府信任感下降** | 5 | 4 | 3 | 2 | 1 |
| **8.民众上访/抗议** | 5 | 4 | 3 | 2 | 1 |

**H14.为了解决垃圾处理的各种难题，您在多大程度上愿意做出以下努力？【出示示卡23】**

| | 非常愿意 | 比较愿意 | 不一定 | 不太愿意 | 非常不愿意 | 无法选择 |
|---|---|---|---|---|---|---|
| **1. 经常对家庭生活产生的垃圾进行分类投放** | 5 | 4 | 3 | 2 | 1 | 98 |
| **2. 回收再利用家庭生活物品** | 5 | 4 | 3 | 2 | 1 | 98 |
| **3. 愿意同其他居民一起讨论垃圾分类计划** | 5 | 4 | 3 | 2 | 1 | 98 |
| **4. 愿意作为志愿者定期参与维护环境整洁的公益劳动** | 5 | 4 | 3 | 2 | 1 | 98 |
| **5. 如果政府增加税收能够专门用于改善城市垃圾处理问题，我愿意接受合理增税** | 5 | 4 | 3 | 2 | 1 | 98 |
| **6. 如果有机会，主动与政府、环保组织、专家、垃圾处理方等相关部门交涉垃圾处理问题** | 5 | 4 | 3 | 2 | 1 | 98 |

**H15.您认为哪一方最需要对垃圾分类治理负责任？**

公民个人......1
企业......2
政府......3
公民团体......4
其他（请注明：______）......5

## 环境模块二

**P1a.您认为就我国当前的情况而言，下列各项问题中最重要的是哪个？**

医疗保健 ........................................ 1
教育 ........................................ 2
犯罪 ........................................ 3
环境 ........................................ 4
移民 ........................................ 5
经济 ........................................ 6
恐怖主义 ........................................ 7
贫困 ........................................ 8
以上都不是 ........................................ 9
无法选择 ........................................ 98

**P1b.您认为第二重要的问题是哪个？**

医疗保健 ........................................ 1
教育 ........................................ 2
犯罪 ........................................ 3
环境 ........................................ 4
移民 ........................................ 5
经济 ........................................ 6
恐怖主义 ........................................ 7
贫困 ........................................ 8
以上都不是 ........................................ 9
无法选择 ........................................ 98

**P2.对于以下说法，您在多大程度上同意或不同意？【出示示卡4】**

| | 完全不同意 | 比较不同意 | 无所谓同意不同意 | 比较同意 | 完全同意 | 无法选择 |
|---|---|---|---|---|---|---|
| **1. 私营企业是解决中国经济问题的最好途径** | 1 | 2 | 3 | 4 | 5 | 98 |
| **2. 缩小贫富差距是政府的责任** | 1 | 2 | 3 | 4 | 5 | 98 |
| **3. 中国应限制进口国外产品来保护本国经济** | 1 | 2 | 3 | 4 | 5 | 98 |
| **4. 中国应限制移民来保护本国生活方式** | 1 | 2 | 3 | 4 | 5 | 98 |
| **5. 国际组织从我国政府手里拿走了太多权力** | 1 | 2 | 3 | 4 | 5 | 98 |

**P3a.对于以下选项，您认为目前中国最优先做的事情应该是什么？**

维护国内秩序 ........................................ 1
在政府决策中给公民更多话语权 ........................................ 2
抑制物价上涨 ........................................ 3

保护言论自由 ........................................................................4
无法选择 ..............................................................................98

**P3b.您认为目前中国第二优先做的事情应该是什么？**

维护国内秩序 ........................................................................1
在政府决策中给公民更多话语权 ...............................................2
抑制物价上涨 ........................................................................3
保护言论自由 ........................................................................4
无法选择 ..............................................................................98

**P4b.总的来说，您觉得人们总是可信的，还是在和人们打交道时再小心也不为过？**

人们总是可以信任的 ..............................................................1
人们通常是可以信任的 ...........................................................2
通常，在和人们打交道时再小心也不为过 ..................................3
在和人们打交道时总是再小心也不为过 .....................................4
无法选择 ..............................................................................98

**P4c.总的来说，您认为大多数人是可以信任的还是您在与人交往的时候不得不小心谨慎？**

人们几乎总是可以信任的 ........................................................1
人们经常是可以信任的 ...........................................................2
您经常不得不小心谨慎地应付人 ...............................................3
您几乎总是小心谨慎地应付人 ..................................................4

**P5.请问您对下列机构的信任程度如何？0代表“完全不信任”，10代表“完全信任”，请您从0-10中选择一个合适的数字。**

| | 完全不信任 ← | | | | | | | | | → 完全信任 | |
|---|---|---|---|---|---|---|---|---|---|---|---|
| | **0** | **1** | **2** | **3** | **4** | **5** | **6** | **7** | **8** | **9** | **10** | **无法选择** |
| **1.大学研究中心** | 0 | 1 | 2 | 3 | 4 | 5 | 6 | 7 | 8 | 9 | 10 | 98 |
| **2.新闻媒体** | 0 | 1 | 2 | 3 | 4 | 5 | 6 | 7 | 8 | 9 | 10 | 98 |
| **3.工商企业** | 0 | 1 | 2 | 3 | 4 | 5 | 6 | 7 | 8 | 9 | 10 | 98 |
| **4.全国人民代表大会** | 0 | 1 | 2 | 3 | 4 | 5 | 6 | 7 | 8 | 9 | 10 | 98 |

**P6.总体上说，您对环境问题有多关注？**

完全不关心 ...........................................................................1
比较不关心 ...........................................................................2
说不上关心不关心 ..................................................................3
比较关心 ..............................................................................4
非常关心 ..............................................................................5
无法选择 ..............................................................................98

**P7.以下列举了各类环境问题。您认为哪个问题是中国当前最重要的环境问题？**

空气污染......1
化肥和农药污染......2
水资源短缺......3
水污染......4
核废料......5
生活垃圾处理......6
气候变化......7
转基因食品......8
自然资源枯竭......9
以上都不是......10
无法选择......98

**P8.下列关于全球气候变化的说法哪个最符合您的观点？**

全球气候没有变化......1
全球气候变化主要是由于自然原因......2
全球气候变化的原因，自然和人类的作用各占一半......3
全球气候变化主要是由于人类活动......4
无法选择......98

**P9a.您认为气候变化对全球的影响是好是坏？0表示非常坏，10表示非常好。请您从0-10中选择一个合适的数字。**

| 非常坏 | | | | | | | | | | 非常好 | 无法选择 |
|---|---|---|---|---|---|---|---|---|---|---|---|
| ← | | | | | | | | | | → | |
| 0 | 1 | 2 | 3 | 4 | 5 | 6 | 7 | 8 | 9 | 10 | 98 |

**P9b.您认为气候变化对我国的影响是好是坏？0表示非常坏，10表示非常好。请您从0-10中选择一个合适的数字。**

| 非常坏 | | | | | | | | | | 非常好 | 无法选择 |
|---|---|---|---|---|---|---|---|---|---|---|---|
| ← | | | | | | | | | | → | |
| 0 | 1 | 2 | 3 | 4 | 5 | 6 | 7 | 8 | 9 | 10 | 98 |

**P10.您在多大程度上同意或不同意以下说法？【出示示卡 4】**

| | 完全不同意 | 比较不同意 | 无所谓同意不同意 | 比较同意 | 完全同意 | 无法选择 |
|---|---|---|---|---|---|---|
| **1. 现代科学将会在几乎不改变我们生活方式的同时解决环境问题** | 1 | 2 | 3 | 4 | 5 | 98 |
| **2. 我们对未来的环境状况担忧太多而对当前的物价和就业关注不够** | 1 | 2 | 3 | 4 | 5 | 98 |

| | | | | | | |
|---|---|---|---|---|---|---|
| **3. 在现代生活中，几乎我们做的所有事都对环境有害** | 1 | 2 | 3 | 4 | 5 | 98 |
| **4. 对于人类进步给环境带来的损害，人们的担忧有点过分了** | 1 | 2 | 3 | 4 | 5 | 98 |
| **5. 为了保护环境，中国需要经济增长** | 1 | 2 | 3 | 4 | 5 | 98 |
| **6. 经济增长总是对环境有害** | 1 | 2 | 3 | 4 | 5 | 98 |

**P11a.为了保护环境，您在多大程度上愿意支付更高的价格？【出示示卡24】**

非常愿意……1
比较愿意……2
既非愿意也非不愿意……3
不太愿意……4
非常不愿意……5
无法选择……98

**P11b.为了保护环境，您在多大程度上愿意缴纳更高的税？【出示示卡 24】**

非常愿意……1
比较愿意……2
既非愿意也非不愿意……3
不太愿意……4
非常不愿意……5
无法选择……98

**P11c.为了保护环境，您在多大程度上愿意降低生活水平？【出示示卡 24】**

非常愿意……1
比较愿意……2
既非愿意也非不愿意……3
不太愿意……4
非常不愿意……5
无法选择……98

**P11d.为了经济发展，您在多大程度上愿意接受减少国家自然保护区的面积？【出示示卡24】**

非常愿意……1
比较愿意……2
既非愿意也非不愿意……3
不太愿意……4
非常不愿意……5
无法选择……98

**P12.您在多大程度上同意以下说法？【出示示卡4】**

| | 完全不同意 | 比较不同意 | 无所谓同意不同意 | 比较同意 | 完全同意 | 无法选择 |
|---|---|---|---|---|---|---|
| **1. 像我这样的人很难为环境保护做什么** | 1 | 2 | 3 | 4 | 5 | 98 |
| **2. 即使要花费更多的钱和时间，我也要做有利于环境的事** | 1 | 2 | 3 | 4 | 5 | 98 |
| **3. 生活中还有比环境保护更重要的事情要做** | 1 | 2 | 3 | 4 | 5 | 98 |
| **4. 除非大家都做,否则我保护环境的努力就没有意义** | 1 | 2 | 3 | 4 | 5 | 98 |
| **5. 许多关于环境威胁的说法都是夸大其词** | 1 | 2 | 3 | 4 | 5 | 98 |
| **6. 我很难弄清楚我现在的生活方式是对环境有害还是有利** | 1 | 2 | 3 | 4 | 5 | 98 |
| **7. 环境问题直接影响我的日常生活** | 1 | 2 | 3 | 4 | 5 | 98 |

**P13a.您认为汽车尾气造成的空气污染对环境的危害程度是？【出示示卡25】**

对环境极其有害........................................................................1
非常有害..................................................................................2
有些危害..................................................................................3
不是很有害...............................................................................4
完全没有危害............................................................................5
无法选择..................................................................................98

**P13b.您认为工业排放废气造成的空气污染对环境的危害程度是？【出示示卡 25】**

对环境极其有害........................................................................1
非常有害..................................................................................2
有些危害..................................................................................3
不是很有害...............................................................................4
完全没有危害............................................................................5
无法选择..................................................................................98

**P13c.您认为农业生产中使用的农药和化肥对环境的危害程度是？【出示示卡 25】**

对环境极其有害........................................................................1
非常有害..................................................................................2
有些危害..................................................................................3
不是很有害...............................................................................4
完全没有危害............................................................................5
无法选择..................................................................................98

**P13d.您认为中国的江、河、湖泊的污染对环境的危害程度是？【出示示卡 25】**

对环境极其有害........................................................................1
非常有害..................................................................................2
有些危害..................................................................................3
不是很有害...............................................................................4
完全没有危害............................................................................5
无法选择..................................................................................98

**P13e.大体上，您认为由气候变化引起的全球气温升高对环境的危害程度是？【出示示卡 25】**

对环境极其有害........................................................................1
非常有害..................................................................................2
有些危害..................................................................................3
不是很有害...............................................................................4
完全没有危害............................................................................5
无法选择..................................................................................98

**P13f.那么，您认为转基因作物对环境的危害程度是？【出示示卡 25】**

对环境极其有害........................................................................1
非常有害..................................................................................2
有些危害..................................................................................3
不是很有害...............................................................................4
完全没有危害............................................................................5
无法选择..................................................................................98

**P13g.您认为核电站对环境的危害程度是？【出示示卡 25】**

对环境极其有害........................................................................1
非常有害..................................................................................2
有些危害..................................................................................3
不是很有害...............................................................................4
完全没有危害............................................................................5
无法选择..................................................................................98

**P14a.您认为以下哪种方式是能够让中国的工商企业保护环境的最好方式？**

重罚破坏环境的企业..................................................................1
使用税收手段奖励保护环境的企业..............................................2
向企业提供更多的关于保护环境好处的信息和培训............3
无法选择..................................................................................98

**P14b.您认为以下哪种方式是能够让中国的公众及其家庭保护环境的最好方式？**

重罚破坏环境的个人..................................................................1
使用税收手段奖励保护环境的个人..............................................2
向个人提供更多的关于保护环境好处的信息和培训............3
无法选择..................................................................................98

**P15.如果可以的话，您对到大自然进行户外活动的喜欢程度如何？**

一点也不喜欢..........................................................................1
有一点喜欢..............................................................................2
有一些喜欢..............................................................................3
很喜欢......................................................................................4
非常喜欢..................................................................................5
无法选择..................................................................................98

**P16.过去的12个月，您参加户外休闲活动的频率如何？例如远足，看鸟，游泳，滑雪或其他户外活动？**

几乎每天..................................................................................1
一周几次..................................................................................2
一个月几次..............................................................................3
一年几次..................................................................................4
从来不......................................................................................5
无法选择..................................................................................98

**P17a.过去的 12 个月，您乘飞机旅行了几次？往返（包括中转）算作一次。**

记录：[____|____|____]次

【访员注意】998.不知道、999.拒绝回答

**P17b.您坐小汽车或其他机动车辆（包括摩托车、卡车和小货车）一周大约有几个小时，不包括公共交通（如乘坐公共汽车，小巴士和出租车拼车）。**

记录：[____|____|____]小时

【访员注意】998.不知道、999.拒绝回答

**P17c.您一周有几天吃牛肉、羊肉、或包含牛羊肉的食物？**

记录：[____]天

【访员注意】98.不知道、99.拒绝回答

**P18.您家的房子有多少间房间？不包括任何独立的厨房、浴室、车库、阳台、走廊或橱柜。**

记录：[____|____|____]间

【访员注意】998.不知道、999.拒绝回答

**P19a.您经常会特意将玻璃、铝罐、塑料或报纸等进行分类以方便回收吗？**

总是..........................................................................................1
经常..........................................................................................2
有时..........................................................................................3
从不..........................................................................................4
我居住的地方没有回收系统..................................................98

**P19b.您经常会特意为了环境保护而不去购买某些产品吗？**

总是..........................................................................................1
经常..........................................................................................2
有时..........................................................................................3
从不..........................................................................................4

**P20.您是否加入了任何以保护环境为目的的社团？**

是 ........................................................................................................1
否 ........................................................................................................2

**P21.在过去 5 年中，您是否有过以下行动？**

| | 有 | 没有 |
|---|---|---|
| **1.就某个环境问题签署过请愿书** | 1 | 2 |
| **2.给环保团体捐过钱** | 1 | 2 |
| **3.为某个环境问题参加过抗议或示威游行** | 1 | 2 |

**P22.过去的 12 个月，您生活的社区受到以下因素影响的程度如何？【出示示卡 26】**

| | 根本没有影响 | 有一点影响 | 有一些影响 | 有很大影响 | 有非常大的影响 | 无法选择 |
|---|---|---|---|---|---|---|
| **a. 空气污染** | 1 | 2 | 3 | 4 | 5 | 98 |
| **b. 水污染** | 1 | 2 | 3 | 4 | 5 | 98 |
| **c.极端天气（例如强风暴，干旱，洪水，热浪，寒流等）** | 1 | 2 | 3 | 4 | 5 | 98 |

# Z 部分  联系方式

谢谢您参与我们的调查。希望您能告诉我们您的联系方式，以便将来我们还能再联系到您。我们会严格遵守科学研究的伦理及中国有关法律的规定，为您提供的所有信息保密。除了本研究目的之外，不向任何单位和个人泄露，并愿意为此承担法律责任，谢谢您的理解。

**Z1.您的姓名是：** ________________

**Z2.您的手机号码是：**[____|____|____|____|____|____|____|____|____|____|____]

**Z3.您家的固定电话号码是：**[____|____|____|____|____|____|____|____]

**Z3a.**区号是：[____|____|____|____]

**Z4.您的 Email 地址是：** ________________________________________

**Z5.您的邮寄地址是：** _______省______市_____县（区）_____乡镇/街道

____________________________________________

**Z6.邮政编码是：**[____|____|____|____|____|____]

**Z7.如果我们希望与您保持长期联系的话，请问最好的方式是什么？**

拨打手机........................................................................ 1
拨打固定电话.................................................................. 2
发短信............................................................................ 3
发 Email......................................................................... 4
邮寄信件........................................................................ 5
其他（请注明：__________）..................................... 6

**Z8.请记录当前时间：**[____|____]月[____|____]日[____|____]时[____|____]分

**Z9.请访员将访问过程中发现的问题和建议，记录如下：**

[________________________________________________]
[________________________________________________]

【访员注意】记录格式：题号+问题或建议，如：1、A13 受访者不清楚目前的身高，访员估计受访者身高。
